# Identification of genes underlying phenotypic plasticity of wing size via insulin signaling pathway by network-based analysis in *Sogatella furcifera*

**DOI:** 10.1186/s12864-019-5793-z

**Published:** 2019-05-21

**Authors:** Xinlei Gao, Yating Fu, Olugbenga Emmanuel Ajayi, Dongyang Guo, Liqin Zhang, Qingfa Wu

**Affiliations:** 1 Hefei National Laboratory for Physical Sciences at Microscale, School of Life Sciences, University of Science and Technology of China, Hefei, 230027 China; 2 CAS Key Laboratory of Innate Immunity and Chronic Disease, University of Science and Technology of China, Hefei, 230027 China

**Keywords:** Phenotypic plasticity, Wing dimorphism, IIS-PI3K-Akt-FOXO signaling pathway, FOXO ChIP-Seq, Integrated gene network, Wing dimorphism related genes

## Abstract

**Background:**

Phenotypic plasticity is a common and highly adaptive phenomenon where the same genotype produces different phenotypes in response to environmental cues. *Sogatella furcifera*, a migratory pest of rice exhibits wing dimorphism, is a model insect for studying phenotypic plasticity of wing size. The Insullin-PI3K-Akt-FOXO signaling pathway plays a crucial role in the manipulation of wing size in the migratory insects. However, the regulatory mechanism via the pathway involved in wing dimorphism are still unexplored.

**Results:**

Accompanied by special alternative splicing, genes involved in muscle contraction and energy metabolism were highly expressed in the wing hinges of macropters, demonstrating their adaptation for energy-demanding long-distance flights. Based on FOXO ChIP-Seq analysis, a total of 1259 putative target genes were observed in the wing hinges, including wing morph development, flight muscle and energy metabolism genes. An integrated gene interaction network was built by combining four heterogeneous datasets, and the IIS-PI3K-Akt-FOXO pathway was clustered in a divided functional module. In total, 45 genes in the module directly interacting with the IIS-PI3K-Akt-FOXO pathway showed differential expression levels between the two wing hinges, thus are regarded as potential Insulin pathway mediated wing dimorphism related genes (IWDRGs). Of the 45 IWDRGs, 5 were selected for verification by gene knockdown experiments, and played significant roles in the insect wing size regulation.

**Conclusions:**

We provided valuable insights on the genetic basis of wing dimorphism, and also demonstrated that network analysis is a powerful approach to identify new genes regulating wing dimorphic development via insulin signaling pathway in the migratory insect.

**Electronic supplementary material:**

The online version of this article (10.1186/s12864-019-5793-z) contains supplementary material, which is available to authorized users.

## Background

Wing polymorphism is a typical form of phenotypic plasticity commonly observed in insects in which case the same genotype produces two or more distinct alternative phenotypes in response to environmental variations [1–8]. Basically, the long-winged morphs with fully developed wings (macropter or alate) differ from the short-winged morphs with reduced wings (brachypter) or without wings (apter) with respect to flight capability and reproduction [9]. The long-winged morphs have a well-developed flight apparatus, thus are able to escape deteriorating environments and colonize new habitats. However, the short-winged morphs exhibit a fitness trade-off between flight capability and reproduction, therefore, they reproduce earlier and oviposit more than their long-winged counterparts [1, 3]. The ability of a single genotype to exhibit variable morphology or physiology in response to changing environmental conditions is crucial to the adaptation and survival of most life forms [10].

The two rice planthoppers; the white-backed planthopper (WBPH*, Sogatella furcifera*) and the brown planthopper (BPH, *Nilaparvata lugens*), belong to the order Hemiptera and exhibit wing dimorphism. In the two insects, both the long- and short-winged phenotypes are determined by a single genotype in response to variations in environmental cues such as temperature, host quality, population density among others [11, 12]. *S. furcifera* primarily feeds on rice plants, and can migrate over long distances in the temperate and tropical regions of Asia and Australia [13]. The insect sucks plant sap and thus reduces plant vigor. Also, it delays tillering, causes stunting, chlorosis and shriveling grains, and ultimately leads to rice plant death. Moreover, *S. furcifera* transmits devastating rice viruses, including the southern rice black-streaked dwarf virus, which poses an additional threat to rice plants [14]. Both *S. furcifera* and *N. lugens* have five nymphal stages, and their wing buds grow gradually with increasing nymphal stages. However, the long- and short-winged morphs are externally indistinguishable until the adults emerge [15]. *S. furcifera* male adults are typically monomorphic macropterous, whereas the female adults exhibit wing dimorphism [16]. Short-winged morphs are formed under conditions of lower population densities and optimal nutrition, while overcrowding and poor nutrition promote the formation of long-winged morphs. The long-winged morphs possess functional flight apparatus, hence they readily escape adverse habitats and track changing resources, whereas short-winged morphs are flightless, and usually possess higher fecundity than their long-winged counterparts [9, 17]. Wing polymorphism of *S. furcifera* and *N. lugens* therefore contributes significantly to the ecological success of the species in natural and agricultural habitats.

The insulin/insulin-like growth factor signaling (IIS) pathway is an evolutionarily conserved nutrient-sensing pathway that modulates tissue growth and body size in metazoans [18, 19]. The pathway is reportedly associated with the developmental plasticity of eye size in *Drosophila* and of horn size in Rhinoceros beetles [20, 21]. The wing morph switch in *N. lugens* has been reported to be modulated by IIS signaling pathways [22]. Unlike a single insulin receptor (*InR*) gene found in fruit flies, there are two putative homologous genes of *lnR*s; *NlInR1* and *NlInR2,* identified in the *N. lugens*. The *NlInR1* and *NlInR2* have been verified to have distinct functions, as activation of *NlInR1* favors the formation of long-winged morph while *NlInR2* activation supports the growth of the short-winged morph [22]. Also, it has been demonstrated that *NlInR1* acts through the IIS-PI3K-Akt-FOXO signaling cascade, whereas *NlInR2* suppresses the same pathway [22]. The long- and short-winged morphs could be switched up to the fifth-instar nymph, indicating that they could be reversible depending on the activities of *NlInR1* and *NlInR2*, respectively [15]. We identified all the orthologous counterparts of the IIS-PI3K-Akt-FOXO signaling cascade in the *S. furcifera* genome [23], including two insulin receptors; *SfInR1* and *SfInR2*, which are orthologous to *NlInR1* and *NlInR2* in the *N. lugens*. The wing dimorphism controlled antagonistically by two insulin receptors (*InR1* and *InR2*) through the IIS-PI3K-Akt-FOXO signaling pathway was conserved in *S. furcifera* [22]. Therefore, *S. furcifera* and *N. lugens* are ideal models for studying developmental plasticity of wing size in insects [22].

It is worth noting that the target genes regulated by FOXO and the regulatory genes of the IIS-PI3K-Akt-FOXO signaling pathway are still less understood, our study thus investigated the gene profiles between the wing hinges of the two WBPH wing morphs, and discovered the molecular foundations underlying the divergences of morphology and flight related biological processes. The binding motif of FOXO was determined using the ChIP-Seq analysis, and the analysis of the genome-wide putative target genes of FOXO showed an expression of 1259 putative target genes in the wing hinges. Furthermore, an integrated gene interaction network was built to facilitate selection of the candidate genes regulating wing dimorphic development in the insect. Experimental validation of selected genes demonstrated that all the 5 candidate genes play roles in the wing dimorphism. Collectively, our results provide insights on the molecular foundations underlying wing dimorphism and morphological divergence in the migratory insect.

## Results

### Differentially expressed genes observed in wing hinges of the two wing morphs

**Fig. 1:**
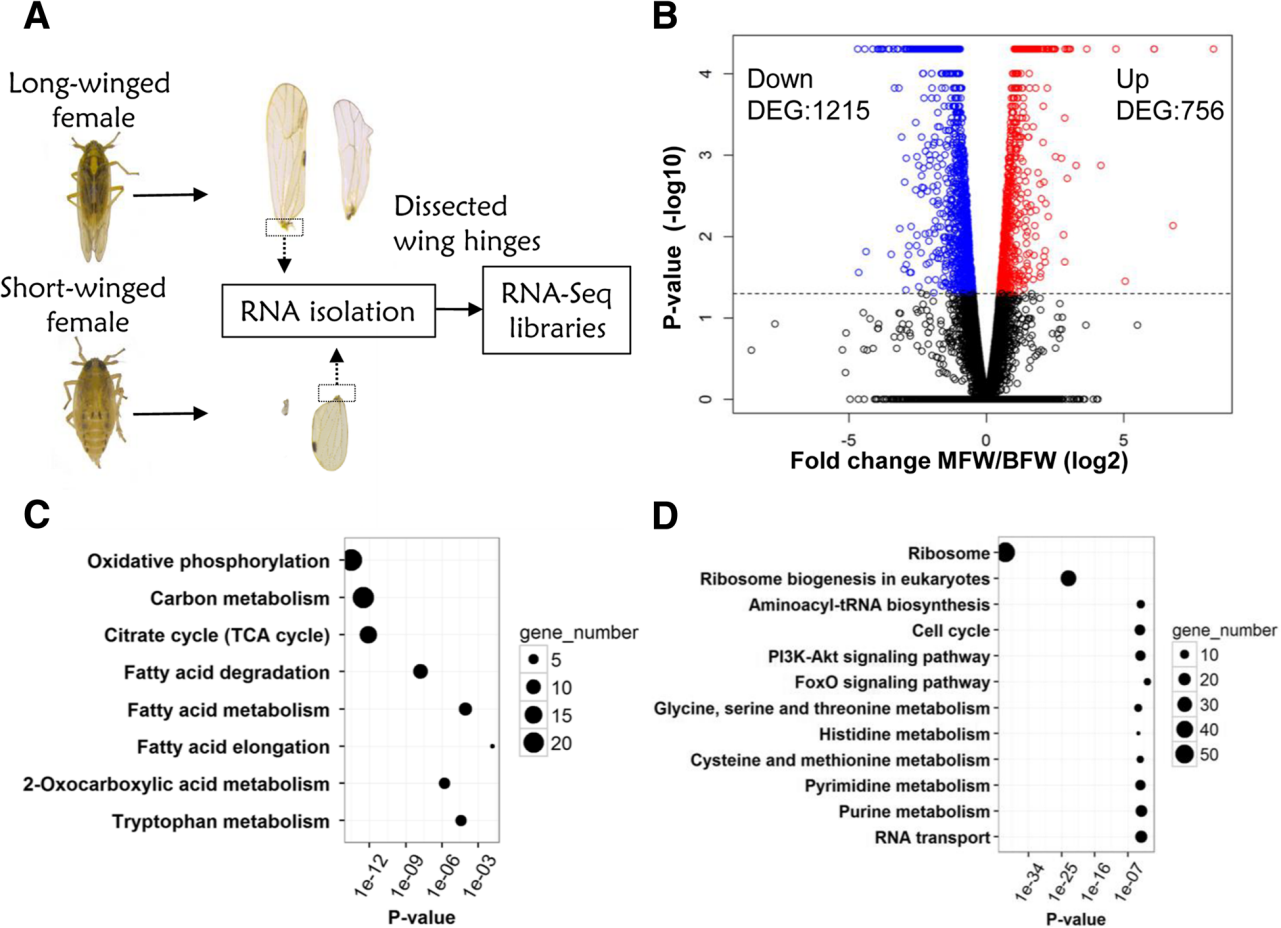
Differentially expressed genes between macropterous female wing hinges (MFW) and brachypterous female wing hinges (BFW). **a** The process of RNA preparation from the wing hinges for RNA-seq analysis was illustrated. **b** The volcano plot of mRNA expressions in the two wing hinges. Plotted along the x-axis is the mean of log_2_ fold-change, and along the y-axis is the negative logarithm of the *p*-values with base 10. Red denotes the 756 up-regulated genes, and blue the 1215 down-regulated genes. The horizontal dot line is the negative logarithm of the *P*-value threshold (*P* = 0.05). **c-d** KEGG pathway enrichment analysis for up-regulated (**c**) and down-regulated DEGs (**d**), respectively, in which the circle size corresponds to gene number

*S. furcifera* male adults are typically monomorphic macropterous, however, the female adults exhibit wing dimorphism. To investigate the gene expression profiles underlying dimorphism in the two wing morphs, the macropterous female wing hinges (MFW) and brachypterous female wing hinges (BFW) of the early adults were studied using RNA-Seq analysis (Fig. 1a and Additional file 1: Table S1). Three biological replicates were performed for each group, and the replicates exhibited good reproducibility, with correlation metrics ranging from 0.84 to 0.98 (Additional file 1: Figure S8). In comparison to BFW, 756 up-regulated differentially expressed genes (DEGs) and 1215 down-regulated DEGs were identified in MFW (Fig. 1b). Gene Ontology (GO) and Kyoto Encyclopedia of Genes and Genomes (KEGG) enrichment analysis revealed that 522 of 756 up-regulated DEGs have defined functions, and among them, 196 (37.5%) were involved in metabolic processes, including tricarboxylic acid cycle and fatty acid metabolism (Fig. 1c). Among the 10 most significantly up-regulated genes (Additional file 1: Table S2), 4 were flight muscle structural component genes, including *flightin*, PDZ and LIM domain protein 7 (*pdlim7*), and two paralogs of troponin C (*TnC*). Flightin is a structural constituent of flight muscle, hence, the significant changes of flight muscle correspond with the higher flight capability of macropters. Similarly, 822 of 1215 down-regulated DEGs have defined functions and are enriched in the categories of translation and ribosome, cell cycle, amino acid and nucleoside metabolic process, alongside PI3K-Akt and FOXO signaling pathways (Fig. 1d). Notably, 5 of the 10 most significantly down-regulated genes encoded uncharacterized proteins (Additional file 1: Table S3).

Our homology-based analyses showed that the *S. furcifera* genome contains all 36 homologs of the principal wing-patterning genes, according to the known wing developmental genes identified in *D. melanogaster*, *N. lugens* and *A. pisum* [24, 25]. These genes participate in diverse aspects of wing development; including wing cell growth, wing hinge cell growth, wing margin differentiation, bristle differentiation and vein position (Additional file 1: Table S4). Also, RNA-Seq analysis revealed that 6 genes were markedly differentially expressed (*P*-value < 0.05), in which 4 were up-regulated and 2 down-regulated in MFW compared to BFW, respectively (Additional file 1: Figure S1). However, in majority of the wing patterning genes, the expression levels between the two wing morphs were not significantly altered.

### Alternative splicing of flight muscle genes identified in the two wing morphs

**Fig. 2:**
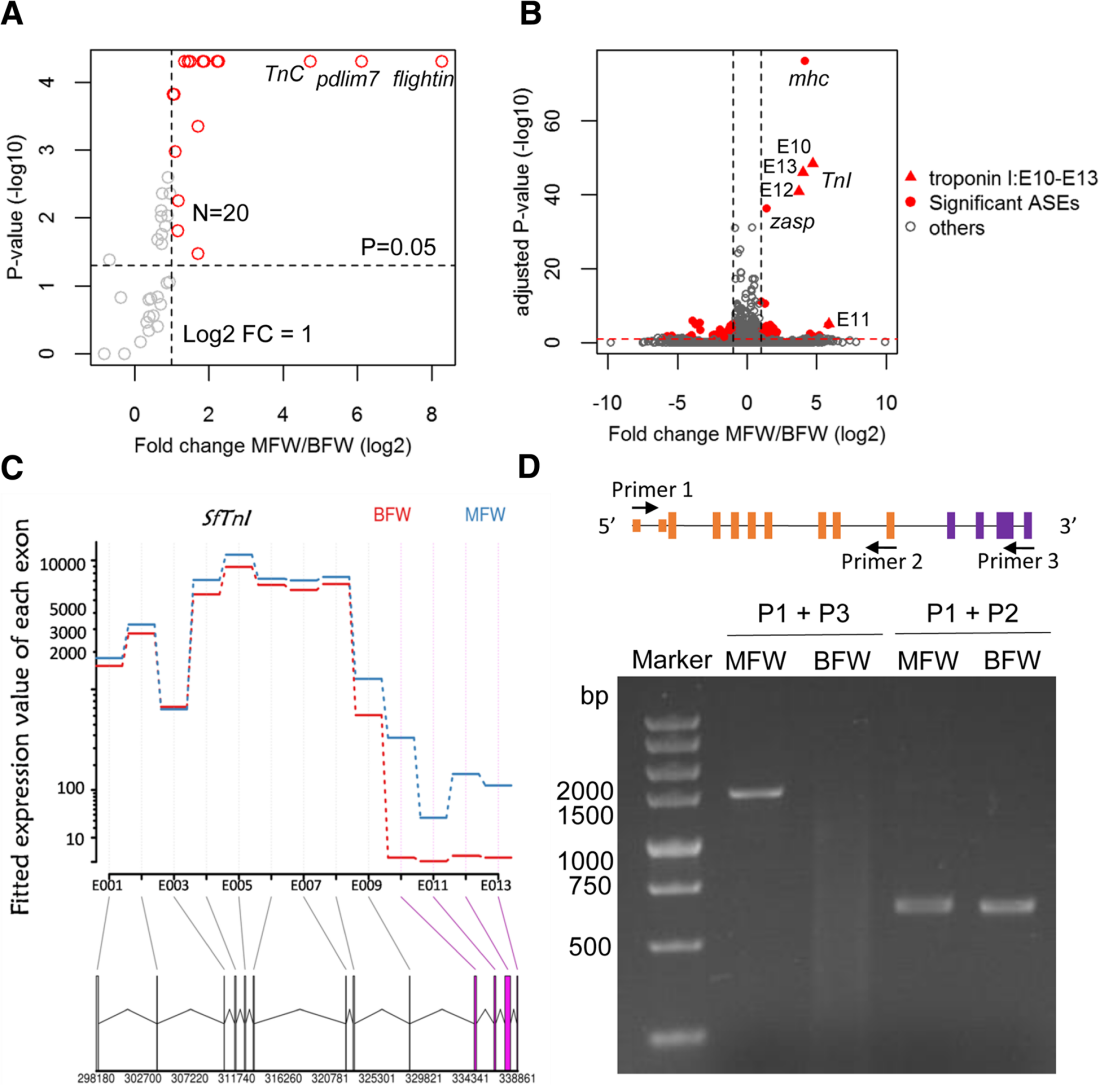
Flight muscle related genes are highly expressed and alternatively spliced in MFW. **a** The volcano plot of the muscle-related genes’ expression profiles. Plotted along the x-axis is log_2_ fold-change of average gene expression levels measured by FPKM values between MFW and BFW, and along the y-axis is the negative logarithm of the *p*-values with base 10. Red circles represent 20 up-regulated genes with *P*-value < 0.05 and log_2_ fold change > 1. The horizontal dot line is the negative logarithm of the *P*-value threshold (P = 0.05). The vertical dot line is the log_2_ fold-change of 1. **b** The volcano plot of all the genes’ differential exon usage. Plotted along the x-axis is the log_2_ fold-change of relative exon usage between MFW and BFW, and along the y-axis is the negative logarithm of the Benjamini-Hochberg (BH) adjusted *p*-values with base 10. Red points are significantly alternatively spliced exons (ASEs) with BH adjusted *P*-value < 0.05 and fold change > 2. The red triangles indicate ASEs in the troponin I gene. The horizontal red dot line is the negative logarithm of the BH adjusted *P*-value threshold (*P* = 0.05). The vertical black dot lines are log_2_ fold-change of − 1 and 1, at the left and right, respectively. **c** Expression levels of each exon of troponin I. Red denotes BFW and blue MFW. Beneath the plot is the gene model of troponin I. **d** The longer and shorter isoforms of troponin I transcripts were verified by RT-PCR. Arrowheads indicate the positions of PCR primers. The exons in yellow represent the common exons between the two isoforms, while the exons in purple represent the exclusive exons of the longer isoform. The primer set of P1 and P3 was used for the longer isoform, while the P1 and P2 set for the shorter isoform

Based on the knowledge of flight muscle genes of *Drosophila* and *N. lugens*, 46 *S. furcifera* flight muscle related genes were identified using homology search (Additional file 1: Table S5). Remarkably, 20 out of the 46 muscle genes were significantly up-regulated in MFW with fold change > 2 and *P*-value < 0.05 (Fig. 2a). These 20 genes included *flightin*, troponin C (*TnC*), *twitchin*, *unc-89*, PDZ and LIM domain proteins among others. *Flightin* and *TnC* also showed higher expression in two other long-winged hemimetabolous insects; *N. lugens* and pea aphid [26, 27]. These results therefore suggest that flight muscle components of flight apparatus are more abundant in MFW than BFW.

Alternative splicing is one of the principal mechanisms that facilitates plasticity by allowing structural and functional differentiation of proteins produced from a single locus, either independently or in addition to changes in expression levels [28, 29]. Using the RNA-Seq data of the two wing morphs, we identified 78 alternatively spliced exons (ASEs) belonging to 60 genes with adjusted *P*-value less than 0.05 and fold change larger than 2. Among them, 35 were included ASEs involving 25 genes and 43 were excluded ASEs involving 35 genes in MFW. Intriguingly, we found that the most significant included ASEs were related to three flight muscle genes, namely; Myosin heavy chain in muscle (*mhc*), troponin I, as well as PDZ and LIM domain protein Zasp (Fig. 2b). The 10 most significant included ASEs in MFW involved 7 WBPH genes. These 7 genes encode Myosin heavy chain in muscle (MHC), troponin I, PDZ and LIM domain protein Zasp, ATP-dependent 6-phosphofructokinase, Putative zm*-*domain protein, Titin and an uncharacterized protein (Additional file 1: Table S6). Notably, 4 of the 7 genes viz.; *mhc*, *titin*, troponin I*,* PDZ and LIM domain protein Zasp are related to flight muscle.

Among them, troponin I possessed 4 included ASEs, while the other three muscle genes each contained a single ASE (Fig. 2b). Troponin I had 13 exons in total, the first 9 exons (exon 1–9) were constitutively spliced into mRNAs in both MFW and BFW, and the last 4 exons (exon 10–13) were exclusively included in MFW (Fig. 2c). Thus, all the 13 exons were spliced into the longer isoform of troponin I in MFW, but the shorter isoform comprising the first 9 exons was the main form in BFW. Correspondingly, the normalized read numbers (reads per million, RPM) spanning the junctions between exons 9–10, 10–11, 11–12 and 12–13 were 20.65, 1.44, 18.26, 7.98 in MFW, in comparison to 0.014, 0, 0.014, 0.014 in BFW, respectively (Additional file 1: Figure S2). RT-PCR analysis confirmed that the longer transcript was exclusively expressed in the macropterous phenotype, while the shorter transcript was expressed in both wing morphs (Fig. 2d). The predicted proteins encoded by the longer transcript and the shorter transcripts were 536 and 205 amino acids in length, respectively. Both the longer and truncated troponin I has a troponin domain identified using InterProScan at 50–179 aa. Multiple sequence alignment was performed to compare troponin I protein sequences from different species, including *N. lugens*, *B. mori*, *A. pisum*, *A. aegypti*, *A. mellifera*, *L. sticticalis*, *H. armigera*, *S. calcitrans*, *P. xylostella*, *D. plexippus*, *H. sapiens* and *M. musculus* (Additional file 1: Figure S3). All these troponin I proteins range from 182 aa to 335 aa and contain one single troponin domain. Multiple sequence alignment revealed that the *S. furcifera* troponin I had the highest similarity with *N. lugens* troponin I comprised of 335 aa. It remains to be investigated whether the two troponin I of different lengths in *S. furcifera* enhance diverse functions in the two morphs. Nevertheless, it is evident that alternative splicing events plays a vital role in flight muscle development of alternative wing morphs.

### The metabolic genes in fatty acid metabolism have higher expression levels in MFW

**Fig. 3:**
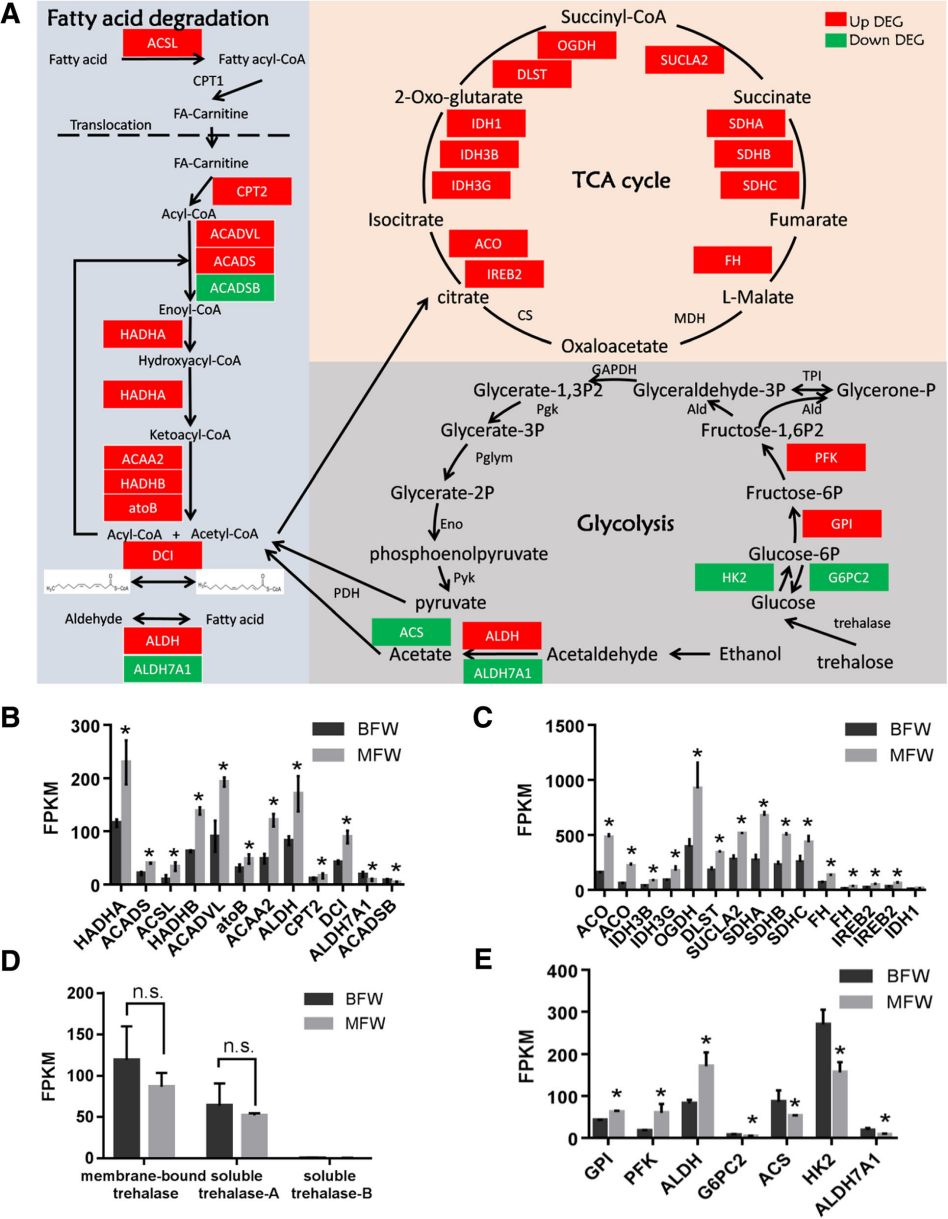
Fatty acid degradation and TCA cycle related genes are up-regulated in MFW. **a** The schematic diagram of energy metabolic pathways including fatty acid degradation, TCA cycle and glycolysis. Genes with red backgrounds are up-regulated DEGs, and genes with green backgrounds are down-regulated DEGs. **b-e** The expressional levels of DEGs in the fatty acid degradation pathway (**b**), the TCA cycle (**c**), trehalases (**d**) and the glycolysis pathway (**e**). * denotes *P* < 0.05, n.s. stands for not significant

Fatty acid and trehalose are both considered main energy resources in insects [30]. In our study, 10 genes in the fatty acid degradation pathway were increased and 2 were decreased in the MFW relative to BFW, indicating the enzymatic activities in fatty acid metabolism might be higher in MFW (Fig. 3a, b). The metabolic intermediate acetyl-CoA generated during fatty acid degradation pathway may enter the TCA cycle for energy release. Eight key enzymes or enzyme complexes are needed to participate in the TCA cycle to completely oxidize acetyl-CoA and generate ATPs. There are also other genes related to the TCA cycle (Additional file 1: Table S7) [31]. In total, 15 *S. furcifera* genes, including 12 genes encoding components of 6 key enzyme complexes and 3 other TCA-related genes, were significantly up-regulated in the MFW compared to BFW (Fig. 3a, c).

Aside fatty acids, trehalose is another vital energy resource with its reserves found in the hemolymph of larvae, pupae and adult insects. Typically, the *S. furcifera* genome has three genes encoding trehalases; one membrane-bound trehalase and two soluble trehalases. Only the membrane-bound trehalase and one of the two soluble trehalase were expressed in *S. furcifera* wing hinges. However, the expression levels of both trehalases were not significantly changed in MFW and BFW but were modestly decreased in the MFW (Fig. 3d).

Trehalase produces glucose which is catalyzed in the glycolysis process- a sequence of ten enzyme-catalyzed reactions that convert glucose into pyruvate. In contrast to the activation of fatty acid degradation in MFW, glycolysis related genes did not show consistent expression changes between MFW and BFW, with 3 genes up-regulated and 4 down-regulated in MFW compared to BFW (Fig. 3a, e). The majority of these enzymes exhibited either insignificant change or undirected change, suggesting that the glycolysis pathway showed no gene expression differences between two wing hinges. Pyruvate is converted to acetyl-CoA by pyruvate dehydrogenase before entering the TCA cycle and there was a slight decrease of pyruvate dehydrogenase in the MFW. We thus inferred that trehalose metabolism is not differentially regulated between MFW and BFW.

### Identification of the genome-wide putative target genes of FOXO

**Fig. 4:**
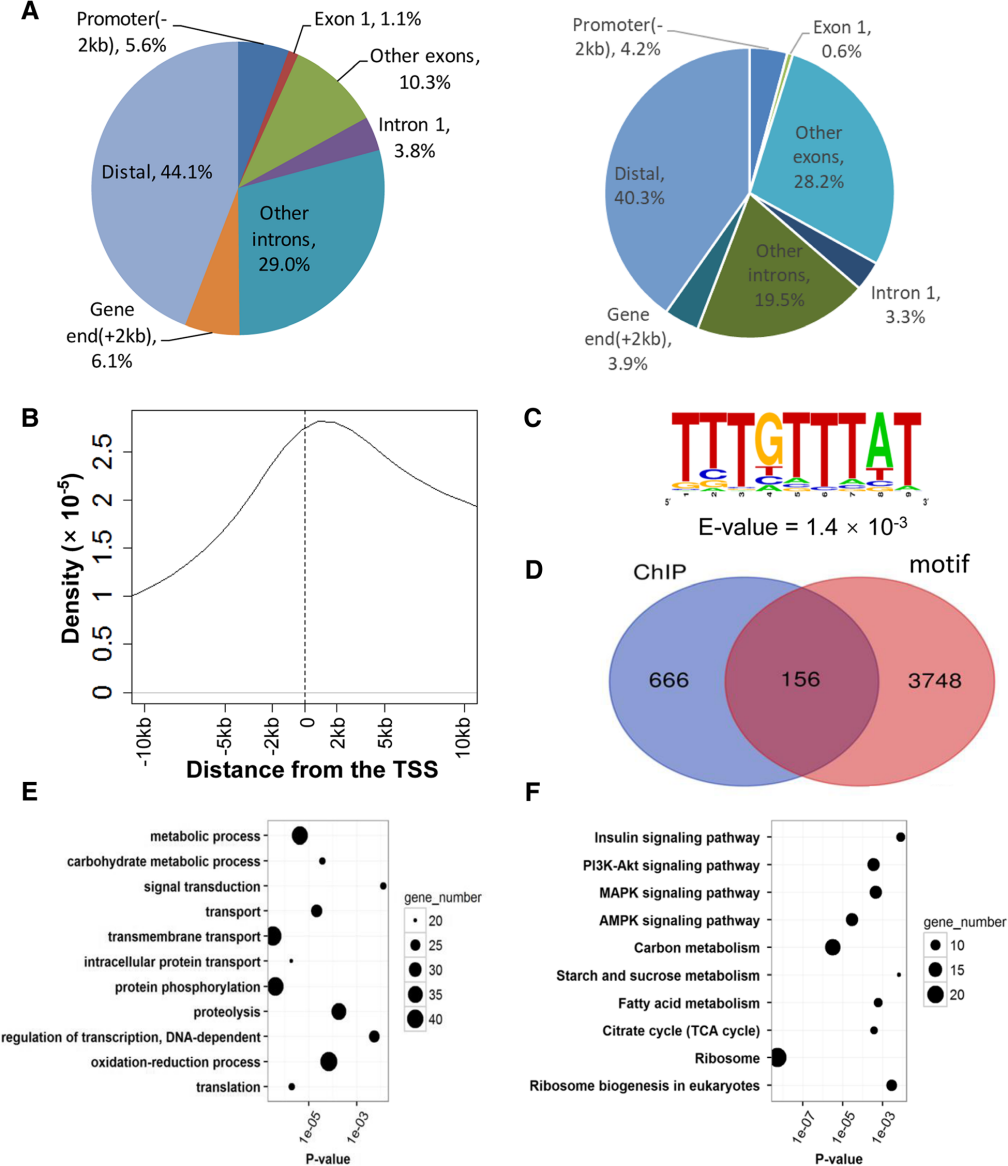
Identification of the genome-wide putative target genes of FOXO. **a** The distribution of FOXO ChIP-seq peaks in different genomic regions (left) and the proportions of the genomic regions in each category (right) were shown. **b** The kernel density plot of distances between peaks and TSSs ranging from − 10 Kb to 10 Kb. **c** The significant FOXO binding motif found in the top 500 binding peaks with smallest q-values by MEME-ChIP. **d** The venn diagram of the putative target genes of FOXO identified from ChIP-seq and genome-wide in silico analysis, respectively. **e-f** The enrichment analysis of the FOXO putative target genes were performed based on GO terms in the biological process category (**e**) and KEGG pathways (**f**), in which the circle size corresponds to gene number

The FOXO protein is the crucial transcriptional factor in regulating the downstream genes responsible for the wing dimorphism. To screen putative target genes in the *S. furcifera* genome, we conducted ChIP-seq of FOXO in cultured *S. furcifera* embryo cells instead of *S. furcifera* tissues, in that using homologous cultured cells will decrease the data heterogeneity. Bioinformatics analysis identified 6692 peaks with 180–400 nt in length (Additional file 1: Figure S4a). Compared with the controls, all peaks have at least 3-fold enrichment, with a median value of 4.485 (Additional file 1: Figure S4b). The peak distributions across the whole genome revealed that the majority of peaks were located in the intergenic regions (44.1%) and introns (32.8%), whereas 11.4% of the peaks were found within exons; and the remaining peaks were located at the upstream and downstream 2 kb flanking gene regions, 5.6% for upstream and 6.1% for downstream, respectively (Fig. 4a). Moreover, the distribution of the peaks was enriched at the nearby regions of the transcription start sites (TSSs), in which 11.7% peaks were located at -2 kb-2 kb regions, and 25.2% peaks were located at -5 kb-5 kb regions (Fig. 4b). Of the 6692 peaks, 822 genes whose TSSs were within 2 kb surrounding the summit of the 6692 peaks were identified as potential FOXO target genes. The binding motifs were identified with MEME-ChIP version 4.12.0 [32] using the top 500 most significant peaks with smallest q-values. The most enriched motif was TTTGTTTAT (Fig. 4c), which was similar to *Drosophila* FOXO binding motif TTGTTTAC, mouse FOXO motif TGTTTAC and *C. elegans* FOXO motif TGTTTGC [33, 34].

To complement the ChIP-Seq analysis, in silico analysis of the genome-wide putative target genes of FOXO was performed. Overall, 41,295 potential FOXO binding sites were identified based on the FOXO motif, and 4182 motifs were located within 2 kb around the TSSs of 3904 genes. Thus, these 3904 genes were regarded as predicted FOXO targets. Aside the 822 genes captured by ChIP-seq analysis, additional 3748 genes were predicted as the FOXO target genes, making a total of 4570 FOXO-regulated genes identified by ChIP-Seq and in silico analysis (Fig. 4d and Additional file 2: Table S8).

There are 10,074 genes expressed in at least one of the two wing morphs. To focus on the potential target genes playing roles in wing dimorphic development, 1259 (12.5% of the expressed genes) putative FOXO target genes with robust expression levels (FPKM ≥10) in at least one of the wing hinges were further analyzed (Additional file 2: Table S8). GO biological process and KEGG pathway enrichment analysis for the 1259 predicted target genes revealed that they are enriched in the metabolic process (carbohydrate metabolic process, tricarboxylic acid cycle and fatty acid metabolism), signal transduction (PI3K-Akt signaling, Insulin signaling and AMPK signaling), transport, translation and transcription regulation (Fig. 4e, f). Of the 1259 potential FOXO targets expressed in at least one wing morph, 11 of 46 flight muscle genes were identified as the target genes (*P*-value = 0.014 by hypergeometric test) (see Additional file 1: Table S9). Also, 9 of 41 fatty acid metabolic genes and 8 of 31 TCA cycle genes were enriched in the predicted FOXO targets with hypergeometric test *P*-value = 0.004 and 0.018, respectively (Additional file 1: Table S10 and S11). KEGG enrichment analysis also revealed that the FOXO putative target genes are enriched in the Insulin signaling and PI3K-Akt signaling pathways. There were 17 putative target genes involved in the both pathways, including AMPK catalytic subunit alpha, *pp2a-b’*, glycogen synthase kinase 3 beta, 14–3-3 epsilon, and *myc*. Akt regulates FOXO activity by phosphorylating and retaining the phosphorylated FOXO in the cytoplasm. However, PP2A-B′ protein can inhibit Akt by dephosphorylating it [35]. The 14–3-3 epsilon antagonizes FOXO by binding to it and preventing its nuclear localization [36]. However, the difference of *foxo* expression levels between MFW and BFW was not significant (FPKM = 29.97 in BFW and 32.90 in MFW, and *P*-value = 0.59 by Cuffdiff2). Further experiments are needed to investigate the regulatory mechanism of these 1259 robustly expressed genes in the MFW and BFW.

### An integrated gene interaction network built for wing dimorphism related genes

Although the IIS-PI3K-Akt-FOXO signaling cascade plays a pivotal role in wing dimorphic development in insects such as brown planthoppers, the gene repertoire, especially those directly interacting with components of the signaling pathway involved in wing dimorphic development, is yet to be explored. To identify new regulatory genes and effectors in wing dimorphic development, an integrated gene interaction network was developed by combining datasets of co-expression interactions, protein-protein interactions, gene functional associated interactions and FOXO-target gene pairs.

Interologs, referred to as conserved protein-protein interactions (PPIs) across species, have been widely applied in many studies on identification of PPIs in non-model organisms [37, 38]. Based on the orthologous group information between *D. melanogaster* and *S. furcifera* [39], we transferred the protein-protein interactions from *Drosophila* proteins to their best hits in *S. furcifera* and obtained 46,381 *S. furcifera* PPIs involving 4889 genes. The gene interactions can also be inferred based on the Gene Ontology semantic similarities because proteins interacting with each other often participate in the same biological process, and co-localize in the common subcellular localizations [40]. These functional associations serve as indirect supporting evidence for interactions between genes [41, 42]. Using the terms of biological process (BP) and cellular component (CC) domains, 42,594 gene pairs are predicted to be functionally associated in *S. furcifera* (see Methods for details).

To infer co-expression relationship between *S. furcifera* genes, a total of 41 RNA-seq libraries were used for weighted gene co-expression network analysis (WGCNA) (Additional file 1: Table S12). To focus on the genes involved in the wing dimorphism and minimize the impact of data heterogeneity on the correlation values between gene pairs, only genes having robust expression levels (FPKM ≥10) in wing hinges and are also expressed in at least 5 samples (10% of 41 samples) were used for further analysis. A total of 4803 genes with robust and variable expression levels among 41 heterogeneous samples were used to construct the WGCNA network (Additional file 1: Supplementary Results) [43, 44]. Based on the topology of the WGCNA network, the topological overlap matrix (TOM) was calculated using the shared neighborhood information of each gene pair, and the topological overlap threshold of 0.3 were further determined to filter the gene pairs. Finally, 92,544 gene pairs with high-confidence co-expression interactions were retrieved, comprising 0.8% of all the possible gene pairs among the 4803 genes.

Taking the three types of gene interactions and 1259 FOXO-target gene pairs together, a multiple heterogeneous network was built, which contained 182,617 interactions involving 7176 genes*.* The logarithm transformed degree distribution (degree refers to the number of connected neighboring nodes of a single node) of the integrated network is in line with the power law with y = 3540.1x^-1.325^ (correlation = 0.973 and R-squared = 0.850) (Additional file 1: Figure S5). Thus, this network exhibits scale free, robust, and error-tolerant characteristics, in consistence with a real biological network [45–47].

**Fig. 5:**
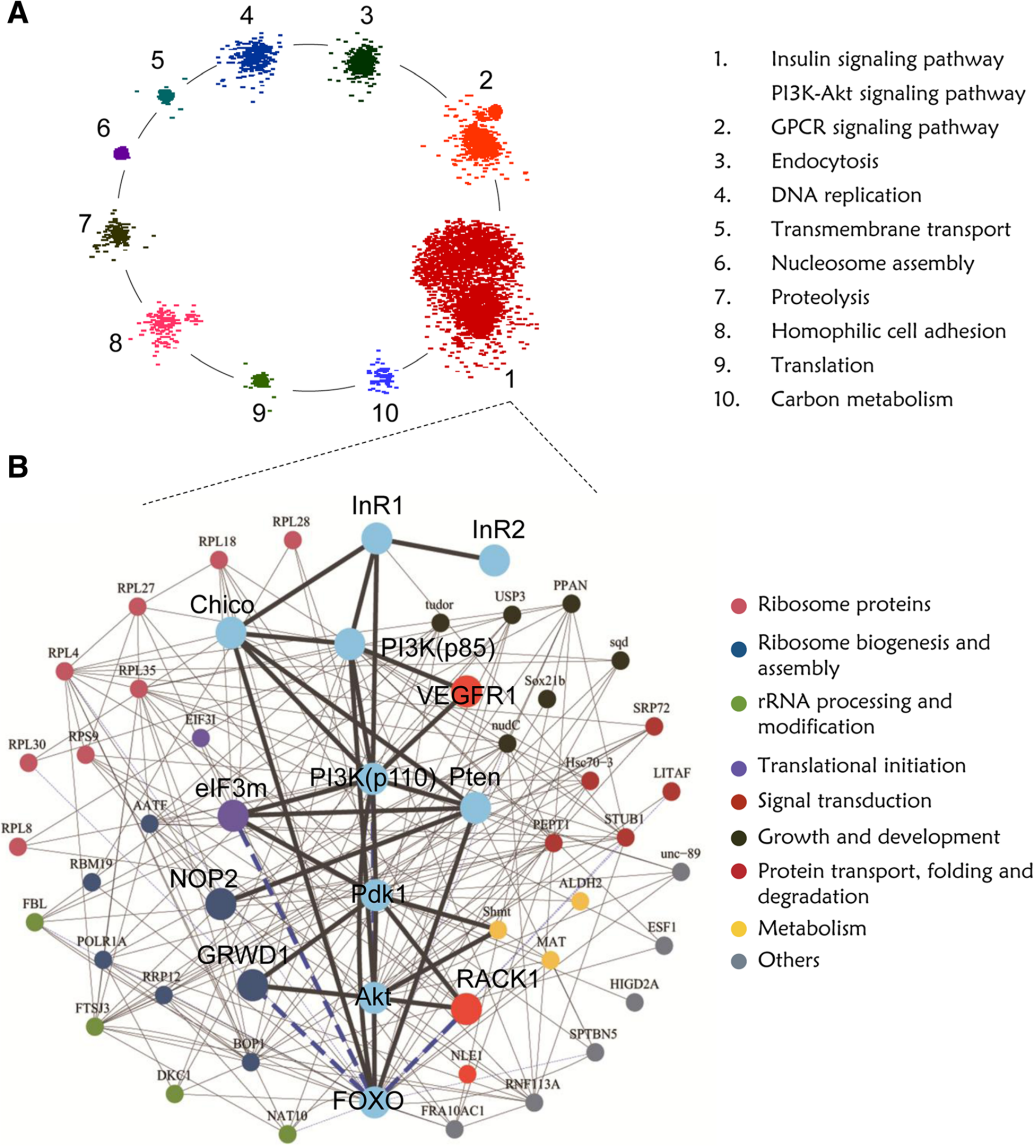
An integrated gene interaction network built for genes related to wing dimorphism. **a** The modular view of the integrated gene interaction network constructed by integrating heterogeneous data together, including protein-protein interactions, co-expression gene pairs, gene pairs with GO semantic similarity and FOXO-target gene pairs. The enrichment analysis of genes in each module were conducted based on GO terms in the biological process category and KEGG pathways. The representatively enriched GO/KEGG terms of each module were shown on the right (*P*-value < 0.0001 by hypergeometric test). **b** The interaction network consisting of the key components of the IIS-PI3K-Akt-FOXO signaling pathway and the 45 IWDRGs. The node colors denote the functional categories of the genes. The bigger nodes in blue represent the components of the IIS-PI3K-Akt signaling pathway, the bigger nodes in other colors denote the IWDRGs for experimental verification. The black solid lines represent gene pairs of protein-protein interaction, co-expression or GO semantic similarity, and the blue dash lines indicate FOXO-target gene pairs

The built integrated network was split into functional modules based on its topological properties using MCL clustering. The 10 largest modules with more than 50 genes were further analyzed, in which the largest module contain 2666 genes and the smallest 58 genes. We classified the 10 modules into functional categories (Fig. 5a and Additional file 1: Table S13). Notably, the module 1 was enriched in signal transduction related genes involved in Insulin signaling (*P* = 7.44 × 10^− 5^, hypergeometric test) and PI3K-Akt signaling pathway (*P* = 1.05 × 10^− 7^). The module 1 contained almost all key components of the IIS-PI3K-Akt-FOXO signaling cascade, including InR1, InR2, IRS, PI3K, Pdk1, Akt and FOXO. Thus, the module 1 was regarded as the Insulin signaling related module. Of note, a total of 300 genes have direct interactions with these components of the IIS-PI3K-Akt-FOXO signaling cascade in the module 1, implying that they might regulate the wing dimorphic development (Additional file 1: Table S14). Interestingly, 45 out of 300 genes showed differential expression between BFW and MFW, thus, these 45 differentially expressed genes are regarded as potential Insulin pathway mediated wing dimorphism related genes (IWDRGs) (Additional file 1: Table S15). Eleven of the 45 IWDRGs were predicted to be FOXO target genes (Additional file 1: Table S16). The interaction network showed that IWDRGs interacted with multiple components of the IIS-PI3K-Akt-FOXO signaling pathway (Fig. 5b).

### Insulin pathway mediated wing dimorphism related genes (IWDRGs) regulates wing size in *S. furcifera*

Among the 45 IWDRGs, 21 (46.7%) were involved in ribosome and translation-related processes, including ribosome biogenesis and assembly, ribosomal proteins, rRNA processing, and translation initiation. The other genes had diverse biological functions, such as growth and developmental processes, regulation of cell cycle and cell proliferation, signal transduction, metabolism, protein folding, protein degradation and so on (Additional file 1: Table S15). Our findings suggest a strong association between translation-related proteins and wing morph development.

**Fig. 6:**
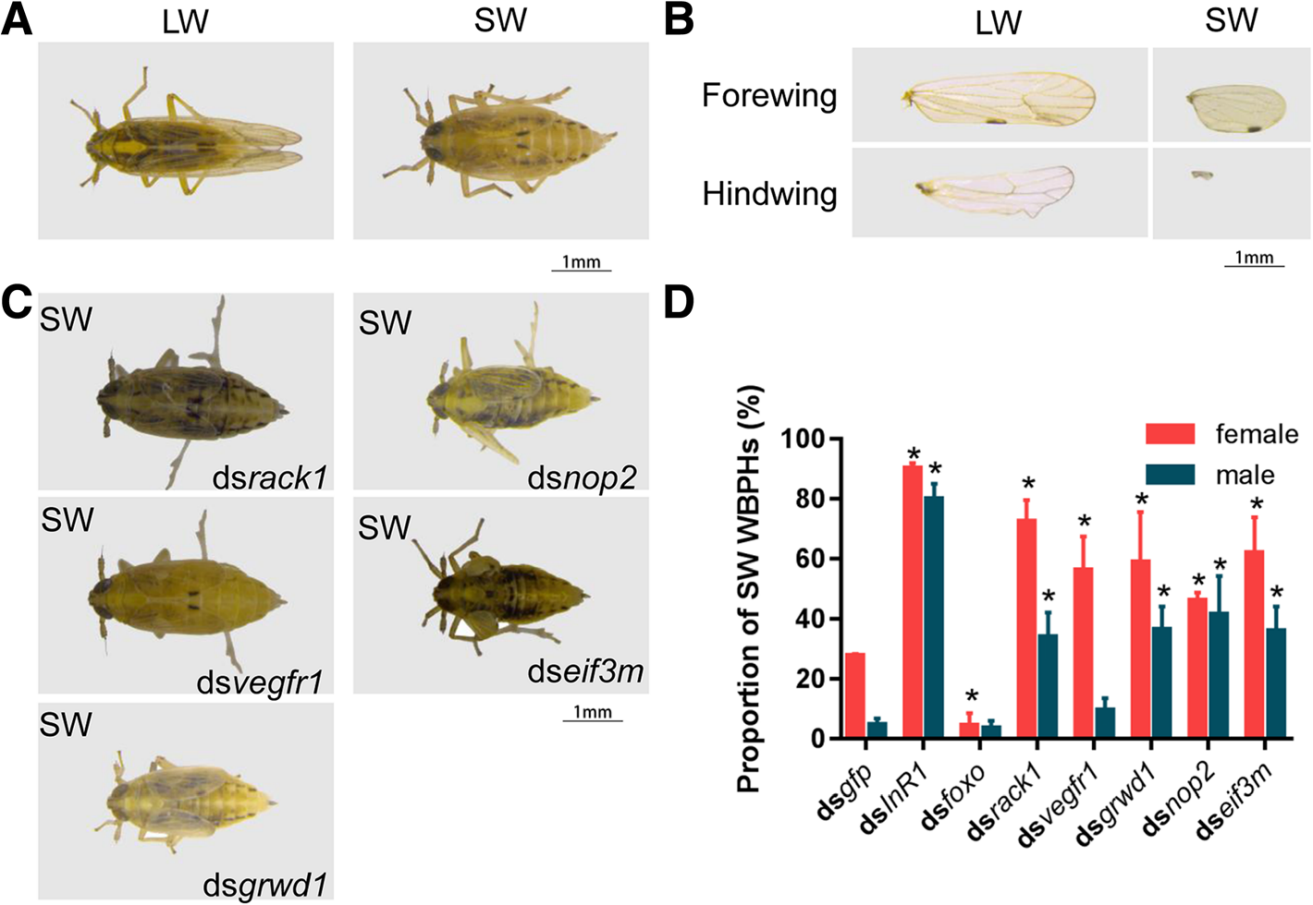
Knockdown of selected IWDRGs verifies their roles in the regulation of wing size in *S. furcifera*. **a, b** The long-winged (LW) and short-winged (SW) *S. furcifera* adults (**a**) and their dissected forewings and hindwings (**b**) served as controls. **c** The representative short-winged (SW) *S. furcifera* adults after gene knockdown by RNAi. **d** The proportions of short-winged (SW) *S. furcifera* after gene knockdown by RNAi. *gfp* knockdown (ds*gfp*) was used as a negative control. Mean and standard error of the mean (S.E.M.) are calculated using three independent experiments. ds*gfp* (*n* = 164 females, 160 males), ds*InR1* (*n* = 83 females, 82 males), ds*foxo* (*n* = 79 females, 74 males), ds*rack1* (*n* = 111 females, 122 males), ds*vegfr1* (*n* = 128 females, 130 males), ds*grwd1* (*n* = 92 females, 108 males), ds*nop2* (*n* = 47 females, 91 males) and ds*eif3m* (*n* = 25 females, 31 males). *P-value < 0.05 by two-sided two-proportions Z-test compared with ds*gfp*

In order to validate the 45 potential IWDRGs predicted by our analysis, 5 candidates viz.; *vegfr1*, *rack1*, *eif3m*, *grwd1*, and *nop2* were selected for experimental verification of their effects on wing dimorphic development (Fig. 5b). The dsRNA of each gene was injected into the 3^rd^-instar *S. furcifera* nymphs to knockdown the gene. Thereafter, the injected nymphs were reared at normal condition and their wing phenotypes monitored till adulthood. The *lnR1* and *foxo* were included as positive controls [22, 48]. Three days after injection, total RNA was extracted from the injected nymphs and qRT-PCR analysis showed that more than 80% transcripts were efficiently knocked down (Additional file 1: Figure S6). Remarkably, knockdown of *InR1* increased the brachypterous morph rate from 4.5% (ds*gfp*, *n* = 160) to 79.9% (*n* = 82, *P*-value = 1.61 × 10^− 37^ by two-sided two proportions Z-test) in males, and from 27.6% (ds*gfp*, *n* = 164) to 90.1% (*n* = 83, *P*-value = 1.65 × 10^− 19^ by two-sided two proportions Z-test) in females. However, knockdown of *foxo* led to a decrease in the brachypterous morph rate from 4.5 to 3.5% (*n* = 74, *P*-value = 0.98 by two-sided proportions Z-test) in males, and from 27.6 to 4.2% (*n* = 79, *P*-value = 0.0038 by two-sided two proportions Z-test) in females (Fig. 6d). Similarly, the transcripts of 5 IWDRGs were efficiently down-regulated using RNAi (Additional file 1: Figure S6). Basically, knockdown of all 5 IWDRGs led to significant changes of wing size, in males and females (Fig. 6).

**Fig. 7:**
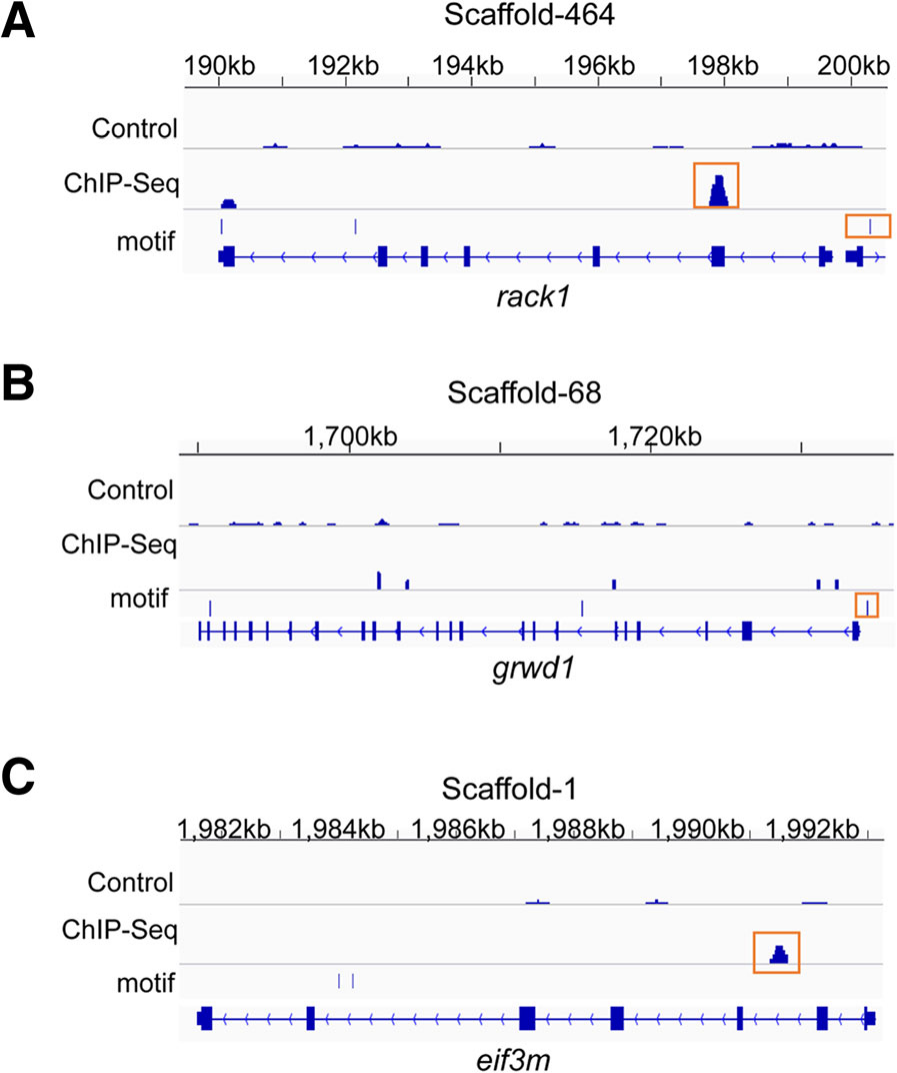
The distribution of FOXO binding sites around *rack1* (**a**), *grwd1* (**b**) and *eif3m* (**c**) genes. Each plot contains four data panels, from top to bottom are ChIP control, FOXO ChIP peaks, FOXO motifs and gene models. The red rectangle frames indicate the FOXO binding signals of either ChIP peak or FOXO binding motif

The *rack1* gene encodes an evolutionary conserved scaffolding protein and its ability to bind a variety of signaling molecules involved in signaling pathways such as cell cycle, apoptosis, cell survival, cell migration and protein translation, are indicative of its role in signal integration [49]. Several lines of evidence cumulate to support the assertion that *rack1* was a FOXO target gene and regulated wing size. First, there was a FOXO peak at 1812 bp with fold enrichment of 6.69 and a predicted binding motif at − 587 bp relative to its TSS (Fig. 7a). Second, RACK1 was found to interact with Pdk1 and Akt of the IIS-PI3K-Akt cascade revealed by our network (Fig. 5b). Third, silencing of *rack1* gene in *S. furcifera* resulted in smaller wings (Fig. 6a, b, c). Knockdown of *rack1* gene raised the brachypterous rate from 4.5 to 33.9% in males (*n* = 122, *P*-value = 9.60 × 10^− 10^ by two-sided two-proportions Z-test), and 27.6 to 72.3% in females (*n* = 111, *P*-value = 2.63 × 10^− 13^ by two-sided two-proportions Z-test) (Fig. 6d).

The *vegfr1* gene mediates phosphorylation of the regulatory subunit of phosphatidylinositol-3-kinase (PI3KR1), leading to activation of PI3K and the downstream Akt signaling pathway [50]. Its knockdown had effects on wing size without altering wing shape (Fig. 6a, b, c). Knockdown of *vegfr1* gene raised the brachypterous rate in males from 4.5 to 9.4% (*n* = 130, *P*-value = 0.23 by two-sided two-proportions Z-test), and in female from 27.6 to 56.1% (*n* = 128, *P*-value = 4.27 × 10^− 5^ by two-sided two-proportions Z-test) (Fig. 6d), implying that *vegfr1* may specifically promote the wing size development in females.

The *grwd1* plays a role in ribosome biogenesis [51]. In a like manner, a FOXO binding motif was predicted at the upstream 631 bp of the *grwd1* gene TSS (Fig. 7b); GRWD1 interacted with Pdk1 and Akt of the IIS-PI3K-Akt signaling pathway (Fig. 5b) and its gene knockdown in *S. furcifera* raised the brachypterous rate from 4.5 to 36.3% in males (*n* = 108, *P*-value = 7.76 × 10^− 11^ by two-sided two-proportions Z-test), and from 27.6 to 58.8% in females (*n* = 92, *P*-value = 7.89 × 10^− 6^ by two-sided two-proportions Z-test) (Fig. 6d).

NOP2 is involved in ribosomal large subunit assembly [52]. Also, in the network, NOP2 interacts with Pten (Fig. 5b) and *s*ilencing of *nop2* increased the brachypterous rate from 4.5 to 41.5% in males (*n* = 91, *P*-value = 7.21 × 10^− 10^ by two-sided two-proportions Z-test), and 27.6 to 46.1% in females (*n* = 47, *P*-value =0.039 by two-sided two-proportions Z-test) (Fig. 6d).

EIF3m (eukaryotic translation initiation factor 3 subunit M) is a part of the eIF-3 complex, which is required for protein synthesis [53]. The eIf-3 complex specifically targets and initiates translation of a subset of mRNAs involved in cell proliferation [54]. Notably, a FOXO ChIP peak was located at the downstream 1675 bp of *eif3m* TSS with fold enrichment at 4.67 (Fig. 7c). The network analysis indicated that eIF3m physically interacts with p110 subunit of PI3K, Pdk1 and Pten (Fig. 5b). Knockdown of *eif3m* gene increased brachypterous rate from 4.5 to 35.9% in males (*n* = 31, *P*-value = 1.01 × 10^− 6^ by two-sided two-proportions Z-test), and 27.6 to 61.9% in females (*n* = 25, *P*-value = 0.0025 by two-sided two-proportions Z-test) (Fig. 6d).

### The multifaceted functions of IWDRGs

The insulin signaling pathway senses nutritional conditions, and coordinates many biological processes, including growth, development, metabolism, protein synthesis, reproduction and lifespan [18, 20, 55–60]. To investigate whether the 5 IWDRGs affect other developmental processes accompanying wing dimorphic development in *S. furcifera*, we also monitored the survival rate, metamorphosis rate and body weights of the insects once these genes were silenced in the 3^rd^-instar nymphs. Basically, *nop2* and *eif3m* had the most significant effects on the growth, development and survival of insects.

**Fig. 8:**
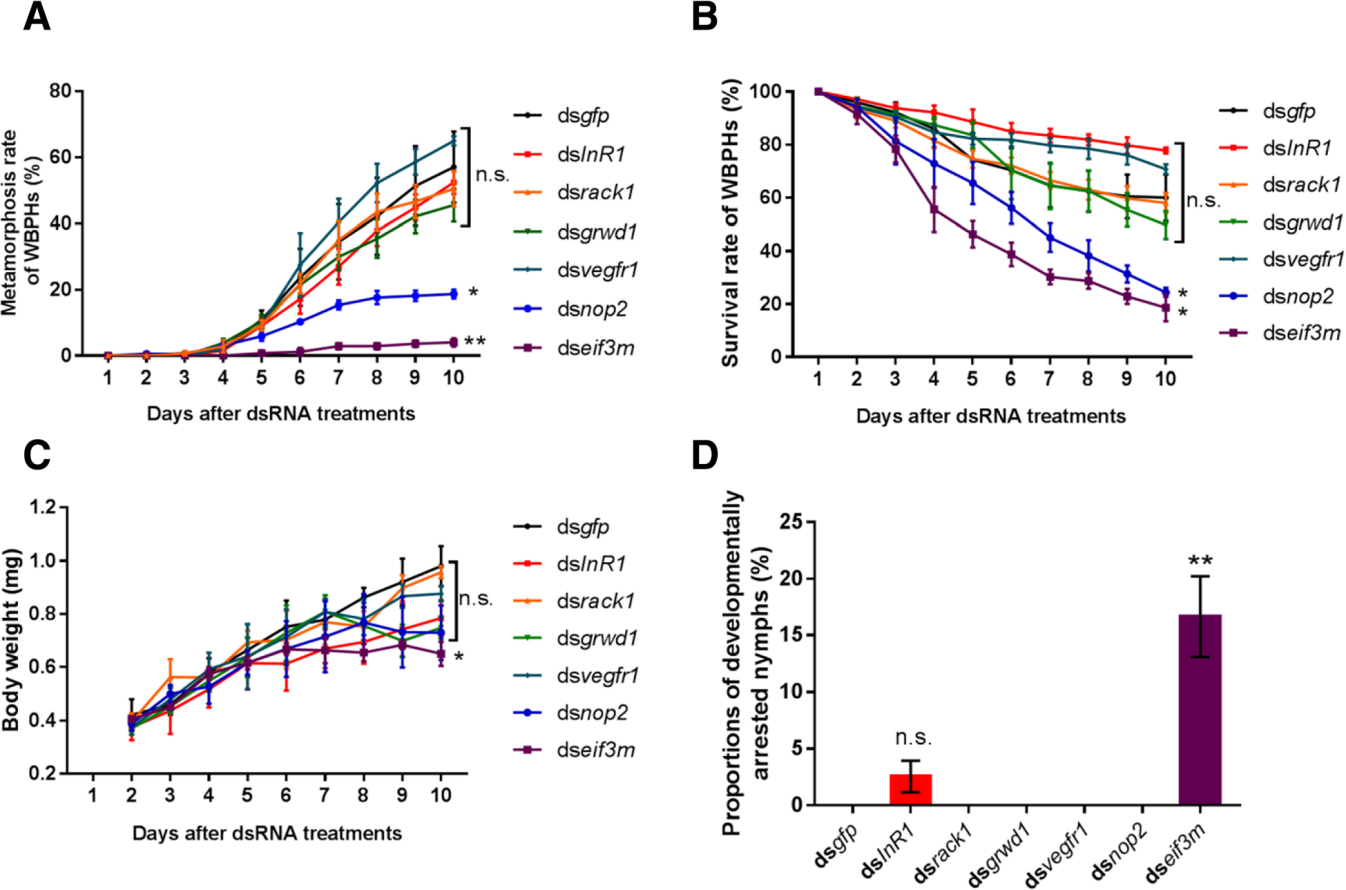
The effects of gene silencing of IWDRGs on the metamorphosis, survival, body weights and developmental arrest of treated nymphs. **a** The metamorphosis rate of WBPH nymphs upon treatment with dsRNAs. **b** The survival curve of WBPH nymphs injected with dsRNAs. **c** The body weight changes of WBPH nymphs after dsRNA treatment. **d** The proportions of developmentally arrested nymphs treated with dsRNAs. Mean and standard error of the mean (S.E.M.) are calculated using three independent experiments. ds*gfp* (*n* = 215), ds*InR1* (*n* = 134), ds*rack1* (*n* = 188), ds*vegfr1* (*n* = 161), ds*grwd1* (*n* = 218), ds*nop2* (*n* = 211) and ds*eif3m* (*n* = 189). * *P* < 0.05, ** *P* < 0.01, n.s. donotes not significant, by two-sided unpaired T-test

First, the metamorphosis rates obtained after silencing of the genes were compared (Fig. 8a). In the control ds*gfp* group, 57.1% of the treated nymphs metamorphosed into adults at the 10^th^ day after dsRNA injection. Knockdown of *InR1, grwd1, vegfr1* and *rack1* caused slight change in the metamorphosis rate, though insignificant; knockdown of *InR1* decreased the metamorphosis rate to 52.4% (*P*-value = 0.70 by two-sided T-test), while the metamorphosis rates were 45.6% (*P*-value = 0.38 by two-sided T-test) with *grwd1* knockdown, 65.0% (*P*-value = 0.51 by two-sided T-test) with *vegfr1 knockdown,* and 50.5% with *rack1* knockdown (*P*-value = 0.61 by two-sided T-test), respectively. However, knockdown of *nop2* and *eif3m* led to significant decrease in the metamorphosis rate. The metamorphosis rate with *nop2* knockdown was 18.7% (*P*-value = 0.02 two-sided T-test), while insects with *eif3m* knockdown had a metamorphosis rate of 4.0% (*P*-value = 0.008 by two-sided T-test).

Furthermore, we investigated the survival rates of the treated insects, and the survival rate of *gfp* knockdown insects was 60% at the 10^th^ day after dsRNA injection (Fig. 8b). Among the experimentally tested genes, only *nop2* and *eif3m* induced significant decrease in the survival rates. Specifically, *nop2* knockdown insects had a survival rate of 24.4% at the 10^th^ day after dsRNA injection (*P*-value = 0.016 by two-sided T-test). Also, *eif3m* gene knockdown decreased the survival rate to 18.6% at the 10^th^ day (*P*-value = 0.015 by two-sided T-test). Aside *nop2* and *eif3m*, the other genes (*InR1*, *vegfr1*, *rack1* and *grwd1*) had survival rates which were not significantly different from that of *gfp* knockdown. With *InR1* knockdown, the survival rate was 77.8% (*P*-value = 0.11 by two-sided T-test); *vegfr1* knockdown yielded a survival rate of 70.7% (*P*-value = 0.30 by two-sided T-test); *rack1* knockdown caused a subtle decrease in the survival rate to 58.0% (*P*-value = 0.84 by two-sided T-test), while *grwd1* knockdown slightly decreased the survival rate to 49.7% (*P*-value = 0.37 by two-sided T-test), all on the 10^th^ day.

Moreover, we monitored the average body weights of treated insects after gene knockdown, and all the IWDRGs caused lower average body weights compared to *gfp* knockdown (Fig. 8c). The average body weights gradually increased with time, and at the 10^th^ day after dsRNA injection, the average weights were 0.98 mg, 0.96 mg, 0.88 mg, 0.78 mg, 0.75 mg, 0.73 mg and 0.65 mg, for knockdown of *gfp*, *rack1*, *vegfr1*, *InR1*, *grwd1*, *nop2*, and *eif3m*, respectively (*P*-value = 0.79, 0.27, 0.13, 0.055, 0.12 and 0.020 for knockdown of *rack1*, *vegfr1*, *InR1*, *grwd1*, *nop2*, and *eif3m*, respectively, by two-sided T-test). Thus, *eif3m* knockdown had the most significant impact on the body weight increase of the insects.

Finally, we characterized the proportions of treated insects that arrested development before metamorphosis due to gene silencing (Fig. 8d). Remarkably, 16.6% of *eif3m* knockdown insects exhibited developmental arrest (*P*-value = 0.0096, by two-sided T-test), as they failed to molt into adults until death. In comparison, knockdown of *gfp*, *rack1*, *vegfr1*, *grwd1* and *nop2* didn’t cause developmental arrest, while 2.5% of the *InR1* knockdown nymphs exhibiting developmental arrest, though not significant (*P*-value = 0.14, by two-sided T-test).

## Discussion

### FOXO orchestrates gene expression in an array of biological processes related to dispersal capability

Wing polymorphism is undeniably one of the evolutionarily successful features found in a wide variety of insect species [61, 62]. The IIS-PI3K-Akt-FOXO signaling pathway is renowned for its crucial role in the manipulation of wing size in *N. lugens* and *S. furcifera* [22, 63]. Basically, the transcription factor FOXO is obliviously the crucial protein, because FOXO activates or suppresses its target genes in response to changes in the upstream IIS-PI3K-Akt signaling pathway. To identify FOXO target genes, we performed ChIP-Seq of FOXO experiment and identified 822 target genes. The FOXO binding motif was inferred using the 500 ChIP-Seq peaks with most significant enrichments. Using the binding motif TTTGTTTAT, 3904 genome-wide potential target genes of FOXO were predicted. However, the 156 overlapping targets between 822 ChIP-Seq targets and 3904 predicted targets represented neither significantly enriched nor depleted (hypergeometric test, *P*-value = 0.66), implying that both methods have limitation to identify the regulatory relationship between FOXO and target genes. Thus, we combined the results from two different methods to infer FOXO-target gene pairs for downstream analysis. In total, there are 4570 putative targets regulated by FOXO across the *S. furcifera* genome, and among them, 1259 target genes are adequately expressed in the wing hinges of the two wing morphs. These FOXO target genes represent a wide variety of functional categories, including 11 IWDRGs, 11 target genes related to flight muscles, 9 target genes related to fatty acid metabolism, and 8 target genes related to TCA cycle. These target genes are closely related to both wing dimorphic development and physiology of wing morphs. This indicates that the IIS-PI3K-Akt-FOXO signaling pathway coordinates gene expression of an array of biological processes, including wing development, flight apparatus and flight energy requirement, all of which influence the dispersal capability of *S. furcifera*.

The gene profiles between the wing hinges of two wing morphs confirmed that many FOXO-regulated genes are differentially expressed between MFW and BFW. The insulin-like peptide is up-regulated while *InR2* is down-regulated in MFW compared to BFW of the *S. furcifera*. Previous studies in *Drosophila* and mammals have unveiled that FOXO can activate the transcription of *InR* by binding its promoter, hence indicating the existence of a feedback loop [64, 65]. In this study, a number of components of the IIS-PI3K-Akt-FOXO signaling pathway, including 14–3-3 epsilon and the protein phosphatase 2A regulatory subunit *pp2a-b’*, were identified as FOXO target genes. Intriguingly, 14–3-3 epsilon and *pp2a-b’* have opposite roles in the manipulation of pathway activity. They are crucial for subcellular localization and transcriptional regulation activity of FOXO. Akt phosphorylates FOXO and prevents it from nuclear localization while PP2A-B′ protein dephosphorylates and inactivates Akt [35]. On the other hand, the 14–3-3 epsilon directly binds to FOXO and retains it in the cytoplasm [36]. Put together, these results suggest that FOXO might regulate the IIS-PI3K-Akt signaling pathway via a complicated feedback loop involving multiple pathway components. Indeed, previous researches in human have revealed that FOXO transcription factors suppress PP2A in the heart, and that FOXO3a induces expression of PI3K catalytic subunit p110alpha in drug-resistant leukemic cells [66, 67]. Thus, the feedback loop may be conserved across mammals and insects. It has also been speculated that the feedback mechanism of FOXO can increase the sensitivity to the signal of environmental nutritional changes and quickly switch to a favorable growth rate [64, 65]. We thus posit that in wing dimorphic insects like *S. furcifera*, the feedback loop might facilitate a rapid response to changes in food quality and nutritional condition in the environment.

In addition to the components of IIS-PI3K-Akt-FOXO signaling pathway, many target genes regulated by FOXO underlying physiological divergence of the two wing morphs showed differential expression between the wing hinges of the two wing morphs. Of note, genes encoding the structural components of flight muscles, such as *flightin*, troponin C, *twitchin* and *unc89*, has abundant accumulations in MFW, and are markedly higher than those in BFW. These flight muscle genes are involved in the construction of essential mechanical flight apparatus and their relative abundance in the MFW correlates with a strong flight capability [68–70]. Our findings on the increasing expression of flight muscle genes in the macropterous morph corroborates those of Xue et al. [26], Brisson et al. [71] and Yang et al. [27] who asserted that flight muscle genes are highly abundant in the flight capable wing forms of *N. lugens* and aphids compared with the flightless ones. The detection of alternatively spliced exons (ASEs) in the two wing morphs further attests to the complexity of the wing development process. The most significant ASEs are related to flight muscle, suggesting that flight muscle genes are expressed as transcript variants in different wing types. For instance, the last four exons of troponin I are identified as ASEs and are specifically expressed in MFW, which probably leads to different features of flight muscle in two wing morphs. Remarkably, flight is one of the most prominent activities performed by insects and requires a high energy turn-over, thus, insect flight involves mobilization, transport and utilization of endogenous energy reserves at extremely high rates [72]. Since fatty acids can produce more energy than trehalose per unit weight, they are more adapted for long distance flights [30]. Compared with BFW, MFW exhibits increased gene expression related to fatty acid metabolism and TCA cycle. Consequently, the usage of energy resources may be similar between *S. furcifera* and *N. lugens*, as fatty acids is the main flight fuel utilized by the latter during their prolonged flight [73, 74].

In our study, gene expression profiles were generated from adult wing hinges instead of nymphal wing buds. Although there is a possibility that the gene expression specificity in the wing dimorphic development might be missing at this stage, however, it is challenging to get the wing bud samples in the WBPH nymphs. First, it’s hard to define long-winged and short-winged morphs in the nymphal stage, and it is also difficult to dissect the wing bud tissues from nymphs as they are too tiny to operate. Second, RNAi knockdown of *lnR1* and *lnR2* genes in WBPH cannot guarantee 100% of the nymphs developing into adults with the desired wing form (Fig. 6d), also, it will probably conceal the effects on IWDRGs due to the close interplays between Insulin pathway genes and IWDRGs. Despite using wing hinges, the DEGs provided insight into the morphological and physiological differences between the two wing morphs and were helpful to narrow down the candidate IWDRGs in the network analysis.

### Network-based analysis is a powerful strategy to identify genes modulating wing dimorphism

In this study, we developed an integrated gene interaction network by combining 4 gene interaction datasets viz.; co-expression interactions, protein-protein interactions, gene functional associated interactions and FOXO-target gene pairs. Our MCL clustering analysis revealed that the constructed network can be divided into many modules based on the topology, using flow simulation to detect a natural grouping of highly connected nodes [75]. Typically, genes involved in the same biological process interact with one another and have higher expressional correlations among them. This usually contributes higher connectedness to these related genes and differentiates the module comprising them from other parts of the network [76]. The module 1 enriched in the components of IIS-PI3K-Akt-FOXO signaling pathway were supposed to contain other essential genes playing roles in wing dimorphic development. In total, 45 IWDRGs directly interact with the components of the IIS-PI3K-Akt-FOXO signaling pathway. These genes showed differential expression between the two wing hinges, and 5 of them were chosen for experimental verification. Knockdown of the 5 IWDRGs by RNAi led to significant changes in wing size, in males and females (Fig. 6). Interestingly, three IWDRGs, *grwd1, eif3m* and *rack1*, were identified as FOXO target genes (Fig. 7). Therefore, the 5 identified IWDRGs are either modulatory genes of the IIS-PI3K-Akt-FOXO signaling pathway or the downstream effectors regulated by FOXO. Our results demonstrate that network-based analysis is a powerful strategy to identify new genes in modulating wing dimorphism.

It is also worth noting that our strategy for the selection of candidate genes in regulating wing dimorphism is extensible. Basically, genes directly interacting with the known components of the IIS-PI3K-Akt-FOXO signaling pathway were given higher priority for experimental verification due to the importance of the signaling pathway in wing dimorphism [22]. Moreover, the 5 IWDRGs were demonstrated to play important roles in wing dimorphic development, thus, they could be classified as expanded components of the IIS-PI3K-Akt-FOXO signaling pathway and used for future network-based analysis. The reproducible and expandable nature of our strategy thus makes it suitable for the identification of more genes modulating wing size in *S. furcifera* and other insects.

## Conclusions

This study unraveled the signatures of gene expression profiles underlying the physiological and morphological differences between the wing hinges of *S. furcifera* female wing morphs. It also provided insights into a complicated FOXO transcriptional regulatory network involved in a diverse range of wing morph related effectors playing roles in wing morph determination, flight muscle and energy metabolism. The constructed *S. furcifera* gene interaction network made by combining multiple heterogeneous datasets, and split into a series of modules, has significant association with different biological pathways. Moreover, the IIS-PI3K-Akt-FOXO cascade related module facilitated the selection of candidate genes playing potential roles in wing dimorphic development, with five of them verified by gene knockdown experiments. Put together, our study provided a better understanding of the genetic basis of wing dimorphism in insects, and also proposed a potent, reliable and extendable network-based analysis method, which can be adopted for candidate genes selection in other relevant biological processes. Similar network-based analysis can also identify new genes involved in other biological processes.

## Methods

### Rearing and dissection of *S. furcifera*

*S. furcifera* was reared at temperature 26 °C and 70% relative humidity on rice seedlings grown in a man-made climate chamber under a photoperiod of 16 h lightness and 8 h darkness. The long-winged and short-winged female adults were collected for dissection once their wing shapes could be identified. The *S. furcifera* wing hinges were dissected under a binocular. First, the *S. furcifera* were washed three times with both 75% ethanol and PBS to reduce microbial contamination from its body surface. Thereafter, the insects were dissected in a droplet of PBS. The forewing and wing hinge as a whole were dissected from the insect body, then the wing hinges at the tip of dissected tissue was isolated using tweezers carefully. The wing hinges were rinsed in fresh PBS buffer and placed in pre-chilled TRIzol reagent (Invitrogen) for total RNA isolation and stored at − 80 °C until use.

### RNA-Seq library preparation and sequencing

Total RNA was extracted from wing hinges of macropterous and brachypterous planthoppers using TRIzol reagent (Invitrogen) following the manufacturer’s instructions. The libraries were thereafter constructed according to described methods [77], and sequenced using the HiSeq X10 platform (Illumina, San Diego, CA, USA). Three biological replicates were performed for each group. The three RNA-seq libraries sample sizes of the macropterous female wing hinges (MFW) were 35, 21 and 29, and the sample sizes of brachypterous female wing hinges (BFW) were 40, 19 and 20, respectively.

### Evaluation of transcriptome expression profiles between wing hinges of macropterous and brachypterous planthopper

Adaptors were removed from the raw paired-end sequencing data using Cutadapt (version 1.3), with adaptor sequence AGATCGGAAGAGCACACGTCTGAACTCCAGTCAC for the first pair and AGATCGGAAGAGCGTCGTGTAGGGAAAGAGTGTAG for the second [78]. Low-quality bases with quality score below 30 were trimmed, and the first 9 nt of each read was also trimmed due to the large bias in GC content at the beginning of reads. After trimming adaptors and low-quality bases, reads shorter than 20 nt were discarded. Clean reads were mapped to the *S. furcifera* genome by STAR (version 2.5.3ab) with the parameters --readFilesCommand zcat --outFilterMultimapNmax 20 --outFilterMismatchNmax 4 --outFilterIntronMotifs RemoveNoncanonical --outSAMstrandField intronMotif --outReadsUnmapped Fastx [79]. Subsequently, Cuffdiff2 v2.2.1 was employed to calculate FPKM (fragments per kilobase of exon per million fragments mapped) for the evaluation of the gene expression levels and differential expression using its default parameters [80]. The threshold for significantly differentially expressed genes was set as *P* value < 0.05. The GO and KEGG enrichment analysis was conducted on the up- and down-regulated differentially expressed genes separately using hypergeometric test.

### Alternative splicing analysis

In order to detect alternatively spliced exons (ASEs), we used DEXSeq version 1.16.10 to determine differential exon usage in the wing hinge RNA-Seq data [81]. First, the python script dexseq_prepare_annotation.py provided by DEXSeq was used to process the gene annotation file into the required format. Next, read number corresponding to each exon in each gene was counted from the alignment results (BAM files) generated by STAR using the python script dexseq_count.py also provided by DEXSeq. Thereafter, DEXSeq was used to estimate size factors and dispersions across different biological samples. It was also employed in calculating the normalized exon-level expression values, fold changes of the normalized exon expression between two wing morphs, *P*-values and Benjamini-Hochberg (BH) adjusted *P*-values needed to estimate the significance of the changes. The exons with adjusted *P*-values less than 0.05 and fold changes larger than 2 were defined as ASEs. The ASEs with high expression in MFW were included ASEs in MFW; conversely, the ASEs with high expression in BFW were excluded ASEs in MFW.

### Polymerase chain reaction (PCR) analysis

Before using RNA for cDNA synthesis, genomic DNA contamination was removed from RNA using Baseline-ZERO™ DNase (Epicentre, Madison, WI, USA). Total RNA was reverse-transcribed into cDNA using the Revert Aid First Strand cDNA Synthesis Kit (Fermentas, Waltham, MA, USA) and random primers (Promega, Madison, WI, USA) following the manufacturer’s instructions. Polymerase Chain Reaction (PCR) was used to detect the longer (L) and shorter (S) isoforms of troponin I with designed primers (Additional file 1: Table S17) and the products were run on an agarose gel.

### FOXO ChIP-Seq libraries preparation and sequencing

ChIP was carried out as described previously, with modifications [82]. Cultured embryo cells were fixed with 1% formaldehyde for 10 min at room temperature. Glycine was added to a final concentration of 0.125 M to stop cross-linking. After 5 min of additional incubation and two washes with ice-cold PBS, cells were re-suspended in SDS lysis buffer (1% (w/v) SDS, 10 mM EDTA, 50 mM Tris-HCl, pH 8.0) containing complete protease inhibitor cocktail, and were incubated for 10 min on ice. Cell extracts were sonicated with a Bioruptor (Diagenode) to obtain up to 200–1000 bp DNA fragments. The supernatant was diluted 1:10 in ChIP dilution buffer (0.01% (w/v) SDS, 1.1% (v/v) Triton X-100, 1.2 mM EDTA, 16.7 mM Tris-HCl, pH 8.1, and 167 mM NaCl) containing protease inhibitors. The chromatin solution was precleared and immunoprecipitated with antibody to FOXO (Cosmo Bio, cat. no. CAC-THU-A-DFOXO). The complexes were eluted in 1% (w/v) SDS and 50 mM NaHCO3, and cross-links were reversed for 6 h at 65 °C. Samples were digested with proteinase K for 1 h at 50 °C, and the DNA was extracted with phenol/chloroform/isoamyl alcohol. Eluted DNA was PCR amplified and the library sequenced thereafter.

### ChIP-Seq analysis

Clean reads were aligned to the *S. furcifera* genome using Bowtie version 1.1.2 with at most 2 mismatches per read, and only uniquely mapped reads were retained for further analysis [83]. The peak-calling was performed by MACS2 version 2.1.0, and the peaks with fold enrichment > 2 were extracted [84]. Genes containing peaks within +/− 2 kb around their transcription start sites (TSSs) were regarded as potential target genes of FOXO. Next, +/− 500 bp around the 500 most significant peaks with smallest q-values were retrieved to identify over-representative motifs using MEME-ChIP version 4.12.0 [32]. Simple repeat sequences were masked using RepeatMasker (http://www.repeatmasker.org) [85] and the sequences with more than 30% of bases masked were discarded from the MEME analysis. The most significant motif with the smallest E-value estimated by MEME was taken as the FOXO binding motif. To complement the ChIP-Seq data, the whole genome was scanned for all possible occurrences of the identified FOXO motif using FIMO version 4.10.2 with a stringent cutoff *P*-value < 1 × 10^− 5^ [86]. The genes containing FOXO motifs within +/− 2 kb around their TSSs were also regarded as potential target genes of FOXO. The target genes predicted by ChIP peaks and FOXO motifs were combined, and the adequately expressed target genes with FPKM > 10 in at least one of the two wing morphs were retrieved. GO and KEGG enrichment analysis was performed on these potential target genes with adequate expression using hypergeometric test.

### Interolog inference of *S. furcifera* based on homologous PPI of *D. melanogaster*

We collected experimentally verified *D. melanogaster* PPI data from multiple public molecular interaction databases including BIOGRID, DroID and Flybase [87–89]. Afterwards, we obtained a total of 106,742 protein-protein interactions, involving 10,026 *D. melanogaster* genes. In order to better capture the gene interactions involving insulin signaling related genes, we utilized the insulin signaling related protein interaction network constructed in *Drosophila* named InsulinNet. The network contains 1807 interactions between 554 proteins [90]. The interologs corresponding to InsulinNet were also added to the homolog-based interaction network. Next, based on the orthologous group information between *D. melanogaster* and *S. furcifera* [39], homologous PPIs in *S. furcifera* were inferred. All the proteins encoded by *D. melanogaster* and *S. furcifera* were aligned to each other using BLASTP. In order to reduce possible false positives, for every interacting *D. melanogaster* protein pair, we only selected the *S. furcifera* ortholog that best matches the *D. melanogaster* protein with the lowest e-value by BLAST. After this screening, 46,381 *S. furcifera* PPIs were retained, involving 4889 genes.

### Co-expression interactions inferred from an array of *S. furcifera* transcriptome expression data

The transcriptome expression data was comprised of an array of 41 *S. furcifera* RNA-Seq data, including 9 datasets of developmental stages, 23 datasets of SRBSDV infected adults, as well as macropterous female adult, brachpterous female adult, macropterous male adult, 3 replicates of wing hinges of macropterous female adults, and 3 replicates of wing hinges of brachypterous female adults (Additional file 1: Table S12). Gene expression levels were measured using FPKM values. In order to focus on the genes involved in wing morph development and reduce noise in gene expression profiles caused by lowly expressed genes, we only retained genes with robust expression levels (FPKM ≥10) in wing hinges and also in at least 5 samples (10% of 41 samples). We further removed the most 10% invariable expressed genes with lowest median absolute deviation across 41 samples from the gene cohorts, because the majority of these genes represent housekeeping genes in all types of samples. The R package WGCNA (version 1.51) [43, 44] was used to perform weighted gene co-expression network analysis. Specifically, Pearson correlation coefficient (PCC) between FPKM values of every two genes was calculated based on expression profiles of *S. furcifera* transcriptome. Thereafter, to ensure both scale-free topology fitness and adequate network connectivity, a set of soft-threshold power values were tested on the original PCC values to select a most appropriate power according to the criteria recommended by WGCNA, and this was consequently decided as 6. By adding a power to the absolute values of the original Pearson correlation coefficients, the algorithm assigned significantly higher weights to gene pairs with higher correlations. The topological overlap matrix (TOM) was calculated, and network modulation was conducted using the ‘blockwiseModules’ function provided by WGCNA R package. We set 0.3 as the topological overlap threshold for high-confidence co-expression interactions. To associate the co-expression network modules with biological pathways, we conducted GO and KEGG enrichment analysis for each module using hypergeometric test with *P*-value < 0.0001.

### Gene functional associated interactions predicted by Gene Ontology semantic similarities

The R package GOstats version 2.40.0 was used to calculate GO semantic similarity for *S. furcifera* gene pairs [91], in the aspects of Biological Process (BP) and Cellular Components (CC), respectively. To determine a reasonable cutoff for interaction prediction, we investigated the distribution of GO similarities of known *D. melanogaster* interacting protein pairs. The average GO BP and CC similarity scores of *D. melanogaster* known PPI data were 0.110 and 0.217, significantly higher than the average score of 0.077 and 0.142 of randomly selected non-interacting *D. melanogaster* protein pairs (*P*-value = 2.00 × 10^− 34^ for GO BP and 2.53 × 10^− 60^ for GO CC, by Student’s T test) (see Additional file 1: Figure S7). In *S. furcifera*, the 99.9th percentile of GO BP and CC semantic similarity were 0.947 and 1, respectively, significantly higher than the average similarity of known PPIs in *Drosophila* (*P* < 2.2 × 10^− 16^ for both GO BP and CC, Student’s T test). Correspondingly, the cut off value of GO BP similarity larger than 0.947 and GO CC similarity equal to 1 were used, and 42,594 gene pairs were predicted to be functionally associated with each other in *S. furcifera*.

### Network integration and network clustering by Markov cluster algorithm

Four types of interactions, including homology protein-protein interactions (PPIs), co-expression interactions, functional interactions inferred from Gene Ontology (GO) semantic similarity and transcriptional regulations between FOXO and its predicted target genes, were integrated into a single comprehensive *S. furcifera* gene interaction network. The topological properties and power law fit was conducted by Cytoscape (v3.3.0) [92]. Next, we used the Markov Cluster Algorithm (MCL) [75] to partition the whole integrated *S. furcifera* gene interaction network into modules, with the inflation parameter I = 2.1. The modules with gene numbers larger than 50 were retained for further analysis. In order to characterize the representative functions of each module, Gene Ontology (GO) and KEGG pathway enrichment analysis were performed for each module individually, with the threshold of *P*-value less than 0.0001.

### dsRNA preparation

dsRNAs were designed by SnapDragon (https://www.flyrnai.org/cgi-bin/RNAi_find_primers.pl) [93] using coding sequences of genes to be knocked down. dsRNAs with length of 300–500 bp and without potential off-target in the *S. furcifera* genome were selected. DNA templates for dsRNA synthesis were amplified with primers containing the T7 RNA polymerase promoter at both ends (Additional file 1: Table S17). The DNA templates were purified by gel extraction and dissolved in nuclease-free water. The concentration of the DNA templates was quantified using Nanodrop. The quality and size of the templates were further checked using electrophoresis in a 1% agarose gel. Only the templates with a single band and correct size could be used for dsRNA synthesis. Subsequently, dsRNAs were synthesized using the T7 transcription Kit (ScriptMAX® Thermo T7 transcription Kit, Toyobo Co., Ltd. Life Science Department, Osaka, Japan) according to the manufacturer’s instructions. The synthesis system was placed at the air bath or PCR instrument at 37-42 °C for 2–4 h. A 1ul DNase I (Pub. No. MAN0012000) was added, reacted at 37 °C for 15 min, after which 1ul EDTA was added at 65 °C for 10 min to terminate the reaction. The dsRNA products were purified by LiCl precipitation and dissolved using nuclease-free water. The concentration of dsRNA was measured by Nanodrop, and the quality and size of dsRNA was further verified using electrophoresis on 1% agarose gel. The dsRNA product was adjusted to 5μg/ul, and centrifuged for 5 min at 12,000 rpm, 4 °C to remove impurities. The dsRNA product was stored at -80 °C.

### dsRNA microinjection into *S. furcifera* nymphs

The 2^nd^- to 3^rd^-instar *S. furcifera* nymphs were collected and anaesthetized with CO_2_. Injection glass needle was processed using the flaming micropipette puller, model P-97 (Sutter instrument, USA) into an appropriate size with the parameters set as heat = 600; pull = 80; vel. = 150; and time = 150. A 10ul dsRNA was added into the microinjection needle, and subsequently inserted into the capillary holder. The parameters of the microinjection instruments (Eppendorf) were set as: injection pressure 1000 pah, injection time 0.3 s, and pressure compensation 10 pah. The quantity of dsRNA was 30-40 nl for 2^nd^*-* to 3^rd^*-*instar nymphs. dsRNA was injected into the nymphs at their external epidermis of thorax between the mesocoxa and the hind coxa. About 100–200 nymphs were used for dsRNA injection for each gene of interest. One day after injection, the nymphs were reared in tissue culture jars, 7 cm in diameter and 10.5 cm in height, and fed on rice seedlings grown in the jars. The jars were sealed with nylon nets with each jar containing at most 100 nymphs. Two days after injection, the survival nymphs were divided 30–40 nymphs per tube into tubes containing rice seedlings. The rice seedlings were renewed every three days. The culture environment was set as: temperature 27 ± 0.5 °C; light: dark 16:8 h and relative humidity 50 ± 5%. Three days after injection, total RNA was extracted from 5 nymphs to examine gene silencing efficiency using qPCR with designed primers (Additional file 1: Table S17). The remaining nymphs were reared until adults to determine the ratios of macropterous and brachpterous adults. Three independent experiments were performed for each gene knockdown.

### Determination of body weight, metamorphosis rate and survival rate

dsRNAs were injected into about 100~200 2^nd^- to 3^rd^- instar nymphs for each gene to be knocked down. After dsRNA treatment for 24 h, we monitored the number of surviving nymphs, determined the average body weights by weighing them in groups using a CP114 precision scale (Ohaus Corporation) until metamorphosis. Thereafter, we recorded the number of newly emerged adults completing metamorphosis and their wing morph phenotypes at a fixed time every day. It should be noted that 24 h after dsRNA treatment was regarded as the first day, and body weights were recorded from the second day. Three independent experiments were performed for each gene knockdown.

## Additional files

Additional file 1: Supplementary results about co-expression interactions, supplementary figures (**Figure S1** to **Figure S8**) and supplementary tables (**Table S1** to **Table S7**, **Table S9** to **Table S17**). (PDF 1189 kb)

Additional file 2: **Table S8.** FOXO potential target genes predicted by ChIP-Seq analysis and in silico prediction. (XLSX 276 kb)

## Abbreviations

ASE: Alternatively spliced exon
BFW: Brachypterous female wing hinge
BP: Biological process
BPH: Brown planthopper
CC: Cellular component
GO: Gene Ontology
IIS: Insulin/insulin-like growth factor signaling
IWDRG: Insulin pathway mediated wing dimorphism related genes
KEGG: Kyoto Encyclopedia of Genes and Genomes
MFW: Macropterous female wing hinge
PPI: Protein-protein interaction
TOM: Topological overlap matrix
TSS: Transcription start site
WBPH: White-backed planthopper
