# Supplementary material for: Identification of genes underlying phenotypic plasticity of wing size via insulin signaling pathway by network-based analysis in Sogatella furcifera
Source: BMC Genomics. 2019 May 21;20:396. doi: 10.1186/s12864-019-5793-z (PMC6528338; doi:10.1186/s12864-019-5793-z)
Supplement: Supplementary file 1 — Supplementary results about co-expression interactions, supplementary figures (Figure S1 to Figure S8) and supplementary tables (Table S1 to Table S7, Table S9 to Table S17). (PDF 1189 kb) [file 12864_2019_5793_MOESM1_ESM.pdf]

Supplementary Results

Co-expression interactions inferred by WGCNA analysis

We adopted weighted gene co-expression network analysis (WGCNA) to detect co-expression interactions between genes. After data processing as elucidated in the Methods, we determined the co-expression weights among the 4,803 genes, and classified them into 9 modules; ranging from 66 genes to 2,005 genes. To investigate the biological relevance of modules and verify the reasonableness of co-expression weights between genes, we related modules to biological pathways. As expected, genes with similar cellular localization and biological functions were preferentially grouped into the same module, to some extent demonstrating the validity and accuracy of gene co-expression measurements. Specifically, a large number of genes in module 1 were integral to the membrane (153 genes, P-value = 6.19×10^{-25} by hypergeometric test), and played a role in transmembrane transport (77 genes, P-value = 5.26×10^{-16}). Genes in the module 2 were found to be located in the nucleus (80 genes, P-value = 1.34×10^{-15}) and cytoplasm (42 genes, P-value = 3.05×10^{-12}), and were related to signal transduction (43 genes, P-value = 3.38×10^{-9}). Genes in module 3 were involved in metabolic process (21 genes, P-value = 3.74×10^{-10}), tricarboxylic acid cycle (6 genes, P-value = 3.72×10^{-9}), and also located on the mitochondrial inner membrane (4 genes, P-value = 2.42×10^{-5}), while genes in module 4 were located on the ribosome (37 genes, P-value = 7.19×10^{-45}) and functioned in translation (38 genes, P-value = 2.66×10^{-45}). It was also observed that

the signal transduction related module (module 2) contained many insulin signalling pathway components such as insulin receptor, PI3K and Pten, suggesting the potential of the co-expression network to recapture the components in biological pathways such as the IIS-PI3K-Akt signaling pathway. Therefore, in order to incorporate the co-expression interactions with other gene interactions, we set a topological overlap threshold of 0.3 to retrieve 92,544 gene pairs with high correlations, comprising 0.8% of all the possible gene pairs among the 4803 genes.

Supplementary Figures

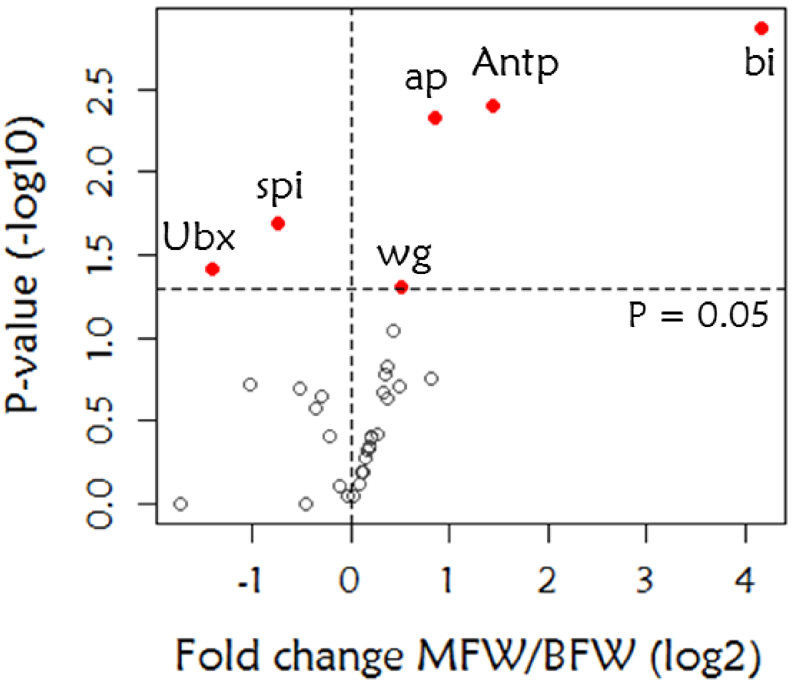

Figure S1. Gene expression changes of wing patterning genes in MFW compared to BFW. The red points indicate significantly differentially expressed genes with P-value < 0.05.

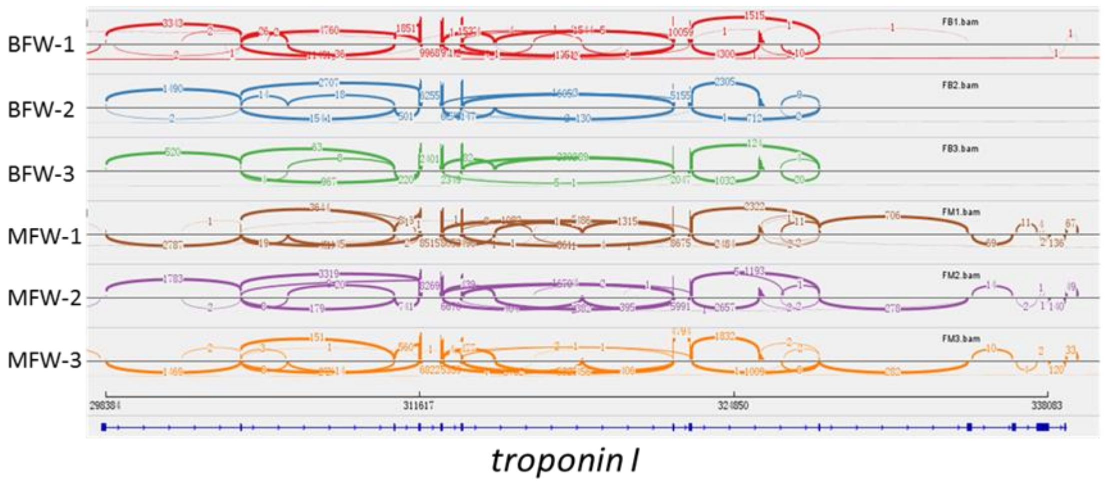

Figure S2. The reads spanning across exons of troponin I. The numbers in the middle of the arc indicate the number of reads spanning two neighboring exons. The last four exons are alternatively spliced exons (ASEs). BFW-1, BFW2 and BFW-3 are three replicates of the brachypterous female wing buds; MFW-1, MFW-2 and MFW-3 are three replicates of the macropterous female wing buds.

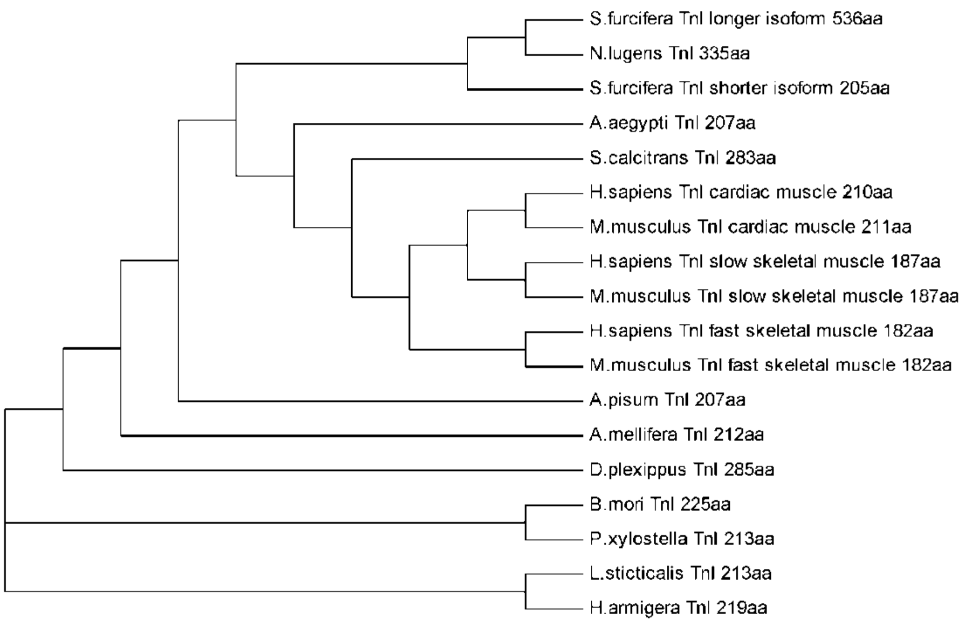

Figure S3. Multiple sequence alignment of troponin I proteins in a variety of species. The Multiple sequence alignment was performed by ClustalX2, and the tree view was produced by MEGA5.05. Analyzed species include *N. lugens* TnI (AGI96984), *A. aegypti* TnI (XP_001661109), *S. calcitrans* TnI (XP_013099761), A. pisum TnI (NP_001313576), *A. mellifera* TnI (NP_001035346), *D. plexippus* TnI (OWR45148), *B. mori* TnI (NP_001037295), *P. xylostella* TnI (XP_011559523), *L. sticticalis* TnI (ABY56688), *H. armigera* TnI (XP_021183945), *H. sapiens* TnI, cardiac muscle (NP_000354), H. sapiens TnI, slow skeletal muscle (NP_003272), *H. sapiens* TnI, fast skeletal muscle (NP_003273), *M. musculus* TnI, cardiac muscle (NP_033432), *M. musculus* TnI, slow skeletal muscle (NP_067442), and *M. musculus* TnI, fast skeletal muscle (NP_033431).

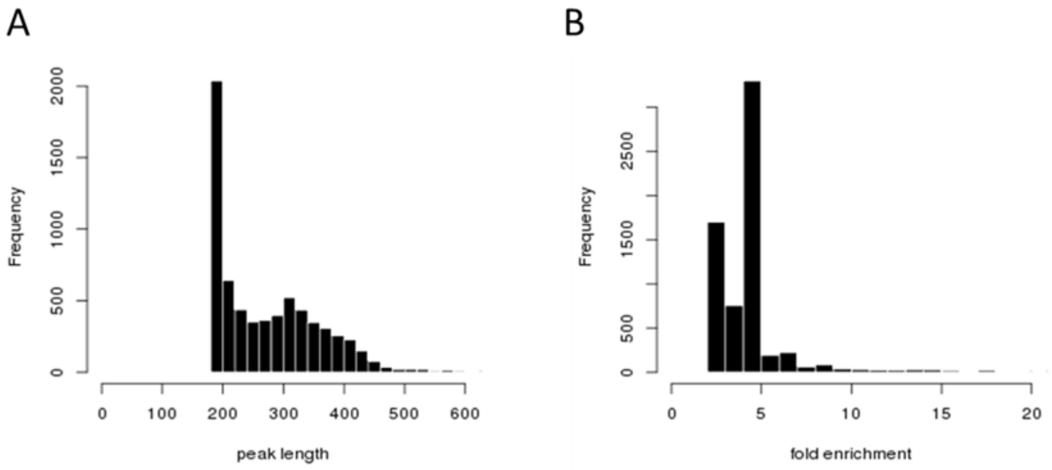

Figure S4. The features of FOXO ChIP peaks. **a.** The length distribution of peaks. **b.** The fold enrichment distribution of peaks.

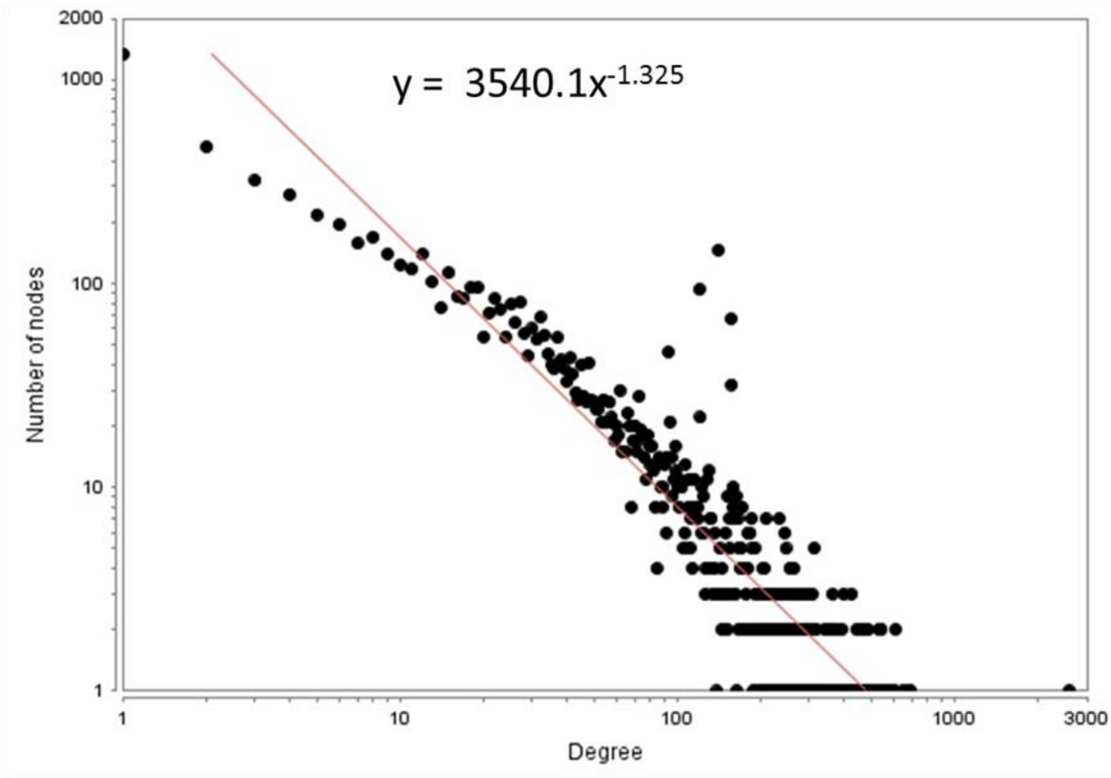

Figure S5. The integrated network satisfies scale-free topology (correlation = 0.973 and R-squared = 0.850).

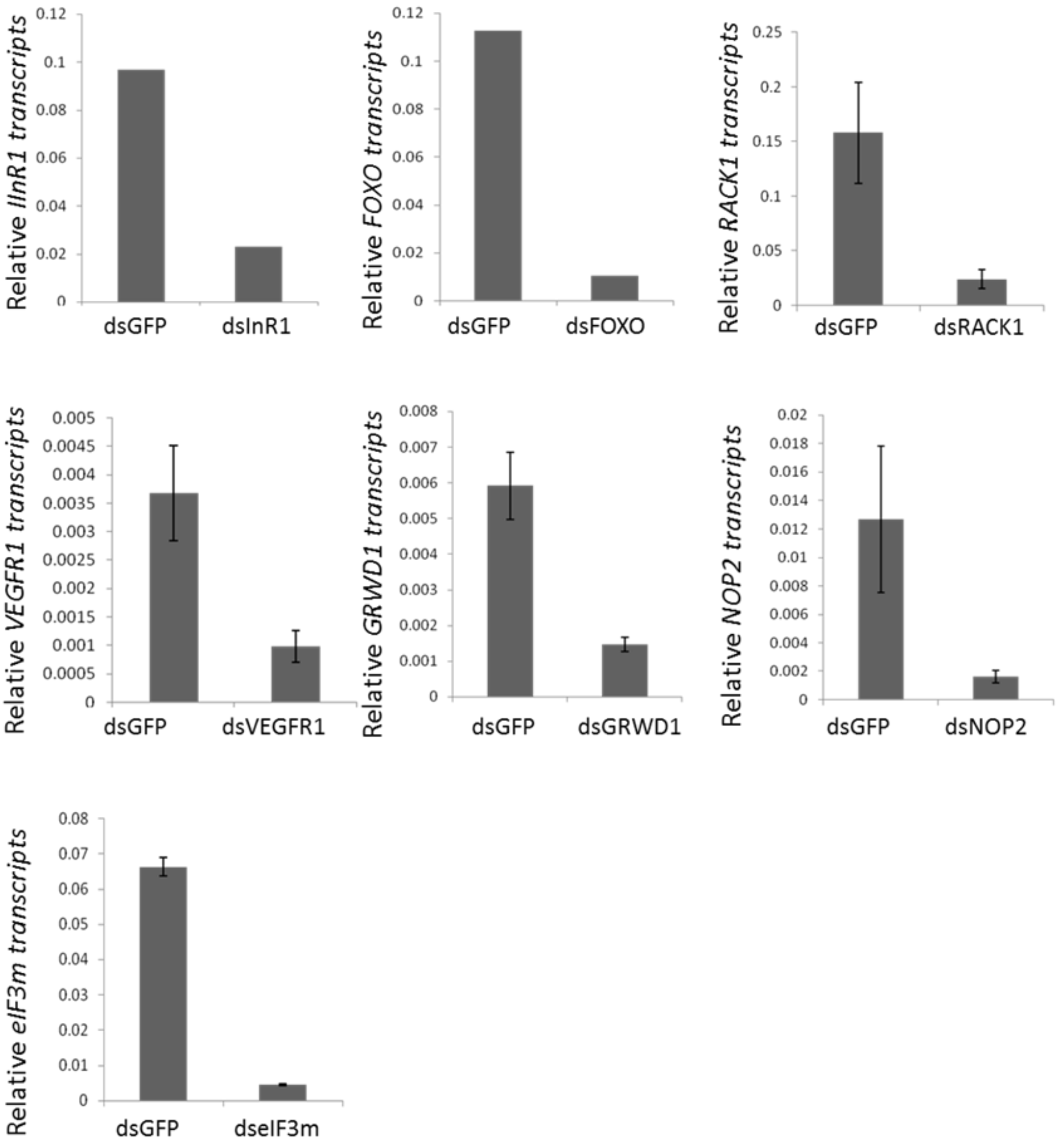

Figure S6. Examination of RNAi efficiency by qRT–PCR analysis. Individual nymphs were pooled (n=5) to extract total RNA after dsRNAs treatments, and cDNA was synthesized with random primers. The relative expression of each gene was normalized to the expression level of RP49 rRNA. Mean+s.e.m. from two experiments.

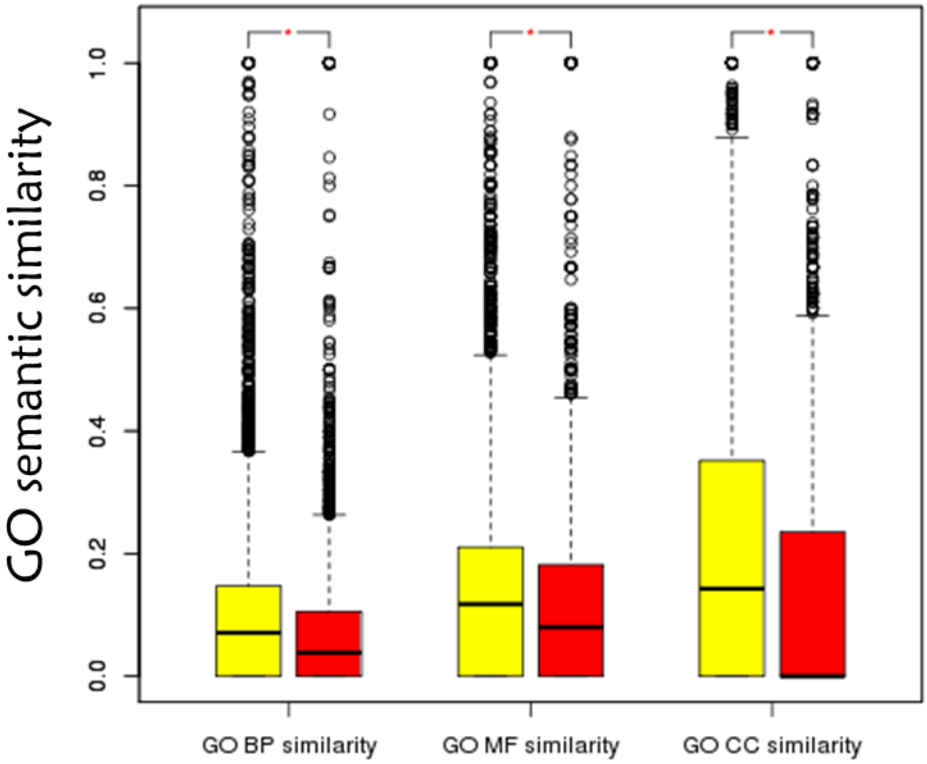

Figure S7. The GO Biological Process (BP), Molecular Function (MF) and Cellular Component (CC) similarity scores of *D. melanogaster* known PPI data were higher than those of randomly selected non-interacting *D. melanogaster* protein pairs. * indicates P-value < 0.05, by Student's T test.

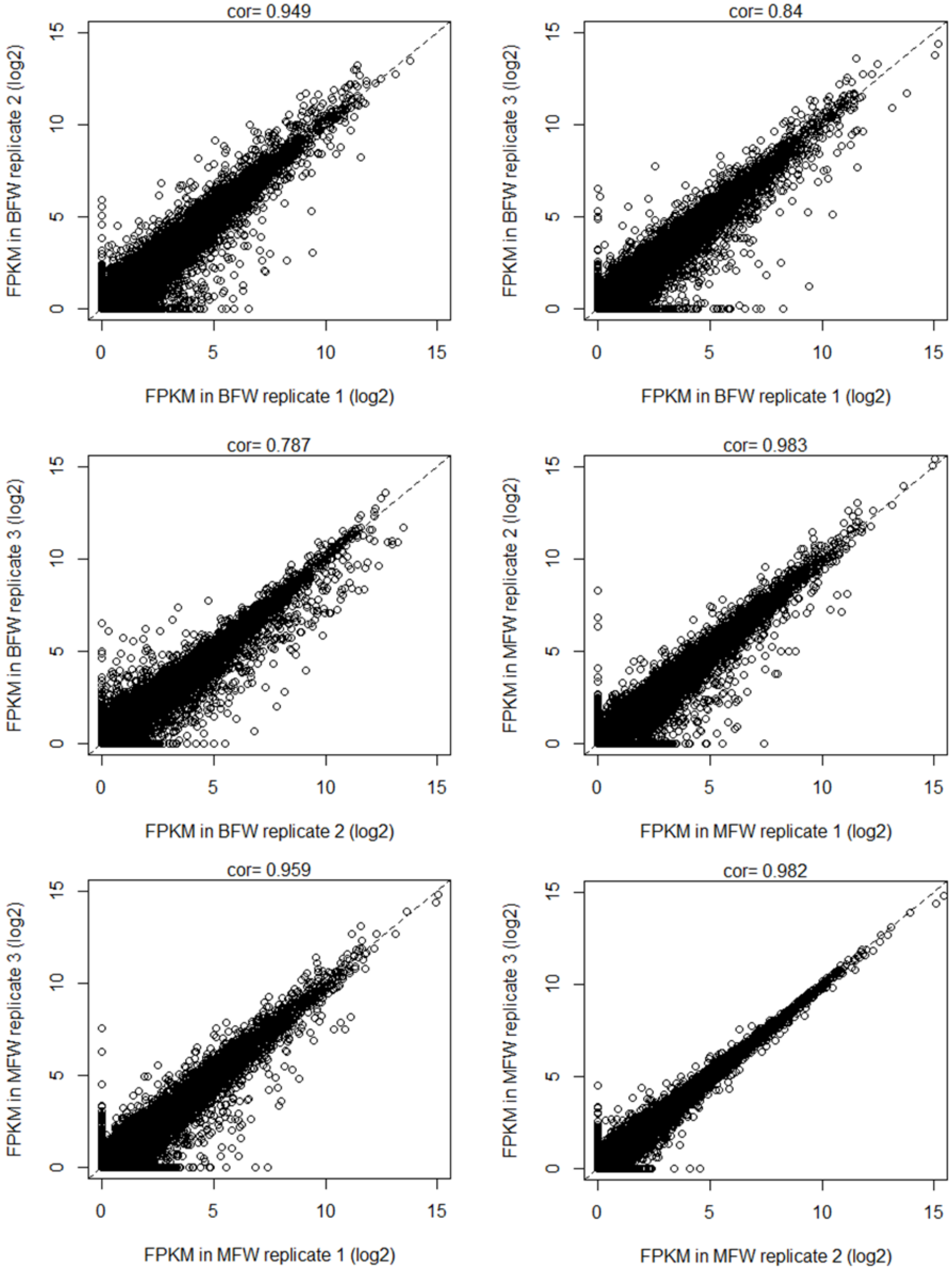

Figure S8. The scatterplots of the expression values (FPKM) between RNA-Seq replicates of MFW and BFW. The correlation metrics are indicated at the top of each plot.

Supplementary Tables

Table S1. QC and statistics of RNA-Seq data

Sample	read pair	read base (G)	Q30 read pair	Q30 read base (G)	Unique mapping rate (%)	Total mapping rate (%)
MFW-1	23093551	6.928065	22057386	5.822426	66.38%	92.45%
MFW-2	19638406	5.891522	19447349	5.381167	65.86%	85.46%
MFW-3	16377336	4.913201	16254691	4.512464	67.05%	86.80%
BFW-1	23359879	7.007964	22384082	5.916622	69.35%	90.72%
BFW-2	18793158	5.637947	18545253	5.15164	69.65%	87.70%
BFW-3	19102332	5.7307	18940430	5.288803	69.27%	87.96%

Table S2. The ten most up-regulated genes in MFW vs. BFW

No.	Gene Name	Gene Annotation	Log2 Fold change	P-value
1	*Sfur-541.2*	Uncharacterized protein	inf	5.00×10^{-5}
2	*Sfnudt1*	7,8-dihydro-8-oxoguanine triphosphatase	inf	5.00×10^{-5}
3	*Sfflightin*	Flightin	8.27	5.00×10^{-5}
4	*Sfpdlim7*	PDZ and LIM domain protein 7	6.11	5.00×10^{-5}
5	*SfTnC*	Troponin C	6.10	5.00×10^{-5}
6	*SfTnC*	Troponin C	4.72	5.00×10^{-5}
7	*Sfur-6.286*	Uncharacterized protein	3.65	5.00×10^{-5}
8	*Sfpkcdelta*	Putative protein kinase C delta type homolog	3.05	5.00×10^{-5}
9	*Sfur-92.68*	Uncharacterized protein	2.99	5.00×10^{-5}
10	*Sfpalladin-like*	Palladin-like	2.50	5.00×10^{-5}

Table S3. The ten most down-regulated genes in MFW vs. BFW

No.	Gene ID	Gene Name	Log2 Fold change	P-value
1	*Sfur-147.41*	Uncharacterized protein	-4.68	5.00×10^{-5}
2	*Sfur-243.17*	Uncharacterized protein	-4.43	5.00×10^{-5}
3	*Sfur-20.103*	Uncharacterized protein	-3.95	5.00×10^{-5}
4	*Sfur-147.40*	Uncharacterized protein	-3.91	5.00×10^{-5}
5	*Sfur-236.19*	Endocuticle structural glycoprotein SgAbd-2	-3.85	5.00×10^{-5}
6	*Sfur-188.11*	Uncharacterized protein	-3.81	5.00×10^{-5}
7	*Sfdefb*	Defensin B	-3.79	5.00×10^{-5}
8	*Sfdat*	Dopamine N-acetyltransferase	-3.56	5.00×10^{-5}
9	*Sfmmp16*	Matrix metalloproteinase-16	-3.53	5.00×10^{-5}
10	*Sffbln2*	Fibulin-2	-3.37	5.00×10^{-5}

Table S4. WBPH orthologs of wing patterning genes.

Function	Gene Name	Gene ID
**Anteroposterior**	*en*	Sfur-20.312
		Sfur-20.315
	hh	Sfur-18.232
	ci	Sfur-78.24
	ptc	Sfur-35.59
	dpp	Sfur-34.12
	dad	Sfur-49.80
	brk	Sfur-384.1
		Sfur-570.6
	salm	Sfur-117.119
	bi	Sfur-15.207
		Sfur-312.13
**Dorsoventral**	*ap*	Sfur-241.22
	notch	Sfur-297.22
		Sfur-318.12
	ser	Sfur-208.4
	wg	Sfur-90.41
	dll	Sfur-20.351
	vg	Sfur-125.25
**Body wall/wing**	*hth*	Sfur-403.20
		Sfur-64.12
	tsh	Sfur-97.37
	nub	Sfur-113.14
	vvl	Sfur-250.11
		Sfur-558.14
**Hox**	*ubx*	Sfur-72.440
**Other**	*spi*	Sfur-22.105
	sna	Sfur-104.15
		Sfur-104.24
	sd	Sfur-21.119
	sc	Sfur-42.117
	exd	Sfur-12.192
	esg	Sfur-3484.1
	ct	Sfur-7.97
	antp	Sfur-72.396
	ac	Sfur-42.117
	abd	Sfur-72.449

Table S5. WBPH orthologs of flight muscle genes.

Function	Gene Name	Gene ID
Thin Filament Proteins	Actin, muscle	Sfur-33.52
	Tropomodulin	Sfur-72.320
	Tropomyosin-1	Sfur-62.34
	Tropomyosin	Sfur-108.6
	Tropomyosin	Sfur-7.153
	Troponin C	Sfur-546.9
	Troponin C	Sfur-296.29
	Troponin C	Sfur-379.2
	Troponin C	Sfur-415.1
	Troponin I	Sfur-490.21
	Troponin T	Sfur-21.40
Thick Filament Proteins	Myosin heavy chain, muscle	Sfur-1.196
	Myosin heavy chain, muscle	Sfur-747.1
	Myosin regulatory light chain 2	Sfur-57.67
	Myosin light chain alkali	Sfur-192.23
	Paramyosin, long form	Sfur-201.15
	Flightin	Sfur-32.201
Z Line Proteins	Muscle LIM protein Mlp84B	Sfur-21.97
	PDZ and LIM domain protein 7	Sfur-39.94
	PDZ and LIM domain protein Zasp	Sfur-106.62
	Alpha-actinin, sarcomeric	Sfur-174.14
	Alpha-actinin, sarcomeric	Sfur-92.101
M Line Proteins	Unc-89	Sfur-22.27
	Unc-89	Sfur-229.29
	Unc-89	Sfur-201.32
	Unc-89	Sfur-1582.1
	Unc-89	Sfur-1071.1
	Unc-89	Sfur-201.34
Sarcomere-Associated Proteins	Talin-1	Sfur-27.94
	Twitchin	Sfur-15.129
	Titin	Sfur-115.24
	Titin	Sfur-3100.1
	Titin	Sfur-245.26
	Titin	Sfur-115.25
	Titin	Sfur-82.16
	Titin	Sfur-81.56

Function	Gene Name	Gene ID
Cytoskeleton-associated Proteins	Spectrin beta chain	Sfur-291.13
	Spectrin beta chain	Sfur-735.1
	CAP-Gly domain-containing linker protein 2	Sfur-67.233
	Paxillin	Sfur-67.158
	Paxillin	Sfur-97.122
	Nesprin-1	Sfur-343.13
	Nesprin-1	Sfur-510.5
	Nesprin-1	Sfur-522.1
Specific in synchronous muscle	Muscle-specific protein 20	Sfur-1.113
	Muscle-specific protein 20	Sfur-10.109

Table S6. Significant included alternatively spliced exons in MFW

Gene Name	Gene Annotation	log2FC	Adjusted P-value
Sfmhc: exon14	Myosin heavy chain, muscle	4.17	9.78×10^{-77}
SfTnI: exon10	Troponin I	4.74	5.18×10^{-49}
SfTnI: exon13	Troponin I	4.02	1.23×10^{-46}
SfTnI: exon12	Troponin I	3.73	1.36×10^{-41}
Sfzasp: exon6	PDZ and LIM domain protein Zasp	1.41	6.11×10^{-37}
Sfpfk: exon5	ATP-dependent 6-phosphofructokinase	1.03	9.20×10^{-12}
Sfur-282.20: exon5	Putative zm-domain protein (Fragment)	1.28	3.96×10^{-11}
SfTnI: exon11	Troponin I	5.89	8.59×10^{-6}
Sftitin: exon19	Titin	5.85	1.74×10^{-5}
Sfur-792.3: exon2	Uncharacterized protein	1.67	1.74×10^{-5}

Table S7. *S. furcifera* genes involved in the TCA cycle and their differential expression.

Genes encoding proteins involved in tricarboxylic acid (TCA) cycle

Enzyme/ Complex	Activity	EC	K number	Isozyme/Subunit		Gene	Differentially expressed (highly expressed in MFW)
CS	citrate synthase	2.3.3.1	K01647	CS		Sfur-1024.1	
						Sfur-399.12	
ACO	aconitate hydratase	4.2.1.3	K01681	ACO		Sfur-192.39	Yes
						Sfur-2604.1	Yes
IDH3	isocitrate dehydrogenase (NAD+)	1.1.1.41	K00030	IDH3	IDH3B	Sfur-33.136	Yes
					IDH3G	Sfur-15.192	Yes
						Sfur-64.41	
OGDHC	oxoglutarate dehydrogenase	1.2.4.2	K00164	E1(OGDH)	OGDH/OGDHL	Sfur-325.12	Yes
						Sfur-230.21	
						Sfur-230.22	
						Sfur-809.1	
					DHTKD1	Sfur-74.195	
	dihydrolipoyllysine-residue succinyltransferase	2.3.1.61	K00658	E2(DLST)		Sfur-32.155	Yes
	dihydrolipoyl dehydrogenase	1.8.1.4	K00382	E3(DLD)		Sfur-555.6	
SUCL	succinate-CoA ligase (GDP-forming)	6.2.1.4	K01899	SUCLG	SUCLG1	Sfur-1112.1	
			K01900		SUCLG2	Sfur-15.161	
	succinate-CoA ligase (ADP-forming)	6.2.1.5	K01899	SUCLA	SUCLG1	Sfur-1112.1	
			K01900		SUCLA2	Sfur-603.13	
						Sfur-72.171	Yes
SDH	succinate dehydrogenase	1.3.5.1	K00234	SDH	SDHA	Sfur-1340.3	
						Sfur-77.64	Yes
			K00235		SDHB	Sfur-50.92	Yes
			K00236		SDHC	Sfur-12.83	Yes
			K00237		SDHD	Sfur-158.63	
FH	fumarate hydratase	4.2.1.2	K01679	FH		Sfur-15099.70	Yes
						Sfur-298.35	Yes
MDH	malate dehydrogenase (mitochondrial)	1.1.1.37	K00026	MDH		Sfur-15.98	

Genes encoding proteins involved in pyruvate and acetyl-CoA metabolism

Enzyme/ Complex	Activity	EC	K number	Isozyme/Subunit		Gene	Differentially expressed (highly expressed in MFW)
PDHC	pyruvate dehydrogenase	1.2.4.1	K00161	E1(PDH)	PDHA1	Sfur-54.111	
						Sfur-78.22	
			K00162		PDHB	Sfur-17.66	
						Sfur-24.144	
	dihydrolipoyllysine-residue acetyltransferase	2.3.1.12	K00627	E2	DLAT	Sfur-204.10	
	E3 binding protein				E3BP	Sfur-6.125	
	dihydrolipoyl dehydrogenase	1.8.1.4	K00382	E3(DLD)		Sfur-555.6	
PC	pyruvate carboxylase	6.4.1.1	K01958	PC		Sfur-125.5	
ACLY	ATP citrate lyase	2.3.3.8	K01648	ACLY		Sfur-272.1	
						Sfur-95.56	

Genes encoding other TCA cycle-related proteins

Enzyme/Complex	Activity	EC	K number	Isozyme/Subunit	Gene	Differentially expressed (highly expressed in MFW)
IREB2	IRE binding protein/aconitate hydratase	4.2.1.3	K01681	IREB2	Sfur-531.2	Yes
					Sfur-622.7	Yes
IDH1	isocitrate dehydrogenase (NADP+)	1.1.1.42	K00031	IDH1	Sfur-225.24	
					Sfur-4.28	Yes
MDH1	malate dehydrogenase (cytosolic)	1.1.1.37	K00025	MDH1	Sfur-123.15	
					Sfur-219.13	
PEPCK	phosphoenolpyruvate carboxykinase (GTP)	4.1.1.32	K01596	PCK/PEPCK	Sfur-368.4	

Table S9. Flight muscle genes identified as FOXO target genes

Gene ID	Gene annotation
Sfur-72.320	Tropomodulin
Sfur-108.6	Tropomyosin
Sfur-490.21	Troponin I
Sfur-1.196	Myosin heavy chain, muscle
Sfur-21.97	Muscle LIM protein Mlp84B
Sfur-174.14	Alpha-actinin, sarcomeric
Sfur-1582.1	Unc-89
Sfur-201.34	Unc-89
Sfur-3100.1	Titin
Sfur-735.1	Spectrin beta chain
Sfur-67.233	CAP-Gly domain-containing linker protein 2

Table S10. Fatty acid metabolic genes identified as FOXO target genes

Gene ID	Gene Annotation
Sfur-12.29	Acetyl-CoA acetyltransferase, cytosolic
Sfur-130.25	Very-long-chain 3-oxoacyl-CoA reductase
Sfur-131.48	Exonuclease 3'-5' domain-containing protein 2
Sfur-134.15	Long-chain-fatty-acid--CoA ligase 3
Sfur-15.189	Fatty acid synthase
Sfur-215.3	Long-chain-fatty-acid--CoA ligase ACSBG2
Sfur-7.19	Carnitine O-palmitoyltransferase 1, liver isoform
Sfur-758.1	Acyl-CoA Delta(11) desaturase
Sfur-88.18	Acyl-CoA desaturase 1

Table S11. TCA cycle genes identified as FOXO target genes

Gene ID	Gene Annotation
Sfur-123.15	Malate dehydrogenase, cytoplasmic
Sfur-15.98	Malate dehydrogenase, mitochondrial
Sfur-192.39	Probable aconitate hydratase, mitochondrial
Sfur-225.24	Isocitrate dehydrogenase [NADP] cytoplasmic
Sfur-24.144	Pyruvate dehydrogenase E1 component subunit beta
Sfur-32.155	Pyruvate dehydrogenase protein X component
Sfur-368.4	Phosphoenolpyruvate carboxykinase [GTP]
Sfur-50.92	Succinate dehydrogenase [ubiquinone] iron-sulfur subunit

Table S12. The 41 *S. furcifera* transcriptome data used for weighted gene co-expression network analysis (WGCNA)

No.	Sample description
1	Embryo
2	Embryo
3	1st instar nymph
4	2nd instar nymph
5	3rd instar nymph
6	4th instar nymph
7	5th instar nymph
8	5-day adult
9	10-day adult
10	adults feeding on viruliferous rice plants with SRBSDV, 9 days
11	adults feeding on viruliferous rice plants with SRBSDV, 16 day
12	viruliferous male adult with high titres of SRBSDV, replicate 1
13	viruliferous male adult with high titres of SRBSDV, replicate 2
14	viruliferous male adult with median titres of SRBSDV, replicate 1
15	viruliferous male adult with median titres of SRBSDV, replicate 2
16	viruliferous male adult with median titres of SRBSDV, replicate 3
17	non-viruliferous male adult, replicate 1
18	non-viruliferous male adult, replicate 2
19	non-viruliferous male adult, replicate 3
20	virus-free male adult, replicate 1
21	virus-free male adult, replicate 2
22	virus-free male adult, replicate 3
23	viruliferous female adult with high titres of SRBSDV
24	viruliferous female adult with median titres of SRBSDV, replicate 1
25	viruliferous female adult with median titres of SRBSDV, replicate 2
26	viruliferous female adult with median titres of SRBSDV, replicate 3
27	non-viruliferous female adult, replicate 1
28	non-viruliferous female adult, replicate 2
29	non-viruliferous female adult, replicate 3
30	virus-free female adult, replicate 1
31	virus-free female adult, replicate 2
32	virus-free female adult, replicate 3
33	macropterous male adults
34	brachypterous female adults
35	macropterous female adults
36	wing buds of brachypterous female adults, replicate 1
37	wing buds of brachypterous female adults, replicate 2
38	wing buds of brachypterous female adults, replicate 3
39	wing buds of macropterous female adults, replicate 1
40	wing buds of macropterous female adults, replicate 2
41	wing buds of macropterous female adults, replicate 3

Table S13. GO and KEGG enrichment analysis for each module of the integrated network (P-value < 0.0001).

Module	GO/KEGG ID	Category	Name	Number of hit genes	Module size	P-value	q-value
**module 1**	GO:0005634	Cellular Component	nucleus	165	2666	3.04×10^{-49}	4.80×10^{-46}
	GO:0006355	Biological Process	regulation of transcription, DNA-dependent	125	2666	4.13×10^{-46}	3.25×10^{-43}
	GO:0005515	Molecular Function	protein binding	438	2666	2.60×10^{-44}	1.37×10^{-41}
	GO:0003700	Molecular Function	sequence-specific DNA binding transcription factor activity	77	2666	2.30×10^{-27}	9.08×10^{-25}
	GO:0005524	Molecular Function	ATP binding	199	2666	2.55×10^{-26}	8.03×10^{-24}
	GO:0007608	Biological Process	sensory perception of smell	33	2666	1.51×10^{-23}	3.41×10^{-21}
	GO:0004984	Molecular Function	olfactory receptor activity	33	2666	1.51×10^{-23}	3.41×10^{-21}
	GO:0000166	Molecular Function	nucleotide binding	87	2666	3.80×10^{-22}	7.48×10^{-20}
	GO:0005549	Molecular Function	odorant binding	33	2666	2.19×10^{-18}	3.83×10^{-16}
	GO:0004812	Molecular Function	aminoacyl-tRNA ligase activity	26	2666	8.56×10^{-16}	1.35×10^{-13}
	GO:0006418	Biological Process	tRNA aminoacylation for protein translation	25	2666	1.67×10^{-15}	2.39×10^{-13}
	GO:0003723	Molecular Function	RNA binding	53	2666	1.53×10^{-14}	2.00×10^{-12}
	GO:0005737	Cellular Component	cytoplasm	56	2666	5.00×10^{-13}	6.06×10^{-11}
	GO:0003824	Molecular Function	catalytic activity	117	2666	9.78×10^{-10}	1.10×10^{-7}
	GO:0006468	Biological Process	protein phosphorylation	73	2666	1.58×10^{-9}	1.54×10^{-7}
	GO:0004672	Molecular Function	protein kinase activity	73	2666	1.58×10^{-9}	1.54×10^{-7}
	GO:0016772	Molecular Function	transferase activity, transferring phosphorus-containing groups	82	2666	1.70×10^{-9}	1.54×10^{-7}
	GO:0008270	Molecular Function	zinc ion binding	147	2666	1.76×10^{-9}	1.54×10^{-7}
	GO:0005488	Molecular Function	binding	73	2666	6.74×10^{-9}	5.59×10^{-7}
	GO:0003676	Molecular Function	nucleic acid binding	219	2666	4.35×10^{-8}	3.42×10^{-6}
	GO:0046872	Molecular Function	metal ion binding	72	2666	1.19×10^{-7}	8.90×10^{-6}
	GO:0008152	Biological Process	metabolic process	68	2666	9.90×10^{-7}	7.09×10^{-5}
	GO:0006396	Biological Process	RNA processing	17	2666	1.68×10^{-6}	0.00012
	GO:0006464	Biological Process	cellular protein modification process	11	2666	2.41×10^{-5}	0.0016
	GO:0055114	Biological Process	oxidation-reduction process	75	2666	3.44×10^{-5}	0.0022
	GO:0043039	Biological Process	tRNA aminoacylation	7	2666	4.37×10^{-5}	0.0026
	GO:0006508	Biological Process	proteolysis	63	2666	5.85×10^{-5}	0.0034
	GO:0006511	Biological Process	ubiquitin-dependent protein catabolic process	18	2666	6.88×10^{-5}	0.0039
	GO:0008168	Molecular Function	methyltransferase activity	15	2666	8.21×10^{-5}	0.0045
	GO:0016876	Molecular Function	ligase activity, forming aminoacyl-tRNA and related compounds	6	2666	9.12×10^{-5}	0.0048
	GO:0016491	Molecular Function	oxidoreductase activity	46	2666	9.84×10^{-5}	0.0050
	ko00970	KEGG	Aminoacyl-tRNA biosynthesis	26	2666	6.29×10^{-15}	2.08×10^{-12}
	ko03040	KEGG	Spliceosome	47	2666	6.48×10^{-14}	1.07×10^{-11}
	ko03018	KEGG	RNA degradation	29	2666	5.45×10^{-13}	6.02×10^{-11}
	ko03008	KEGG	Ribosome biogenesis in eukaryotes	29	2666	1.68×10^{-9}	1.23×10^{-7}
	ko03013	KEGG	RNA transport	42	2666	1.85×10^{-9}	1.23×10^{-7}
	ko04151	KEGG	PI3K-Akt signaling pathway	27	2666	1.05×10^{-7}	4.96×10^{-6}
	ko04150	KEGG	mTOR signaling pathway	15	2666	2.44×10^{-7}	8.96×10^{-6}
	ko04630	KEGG	Jak-STAT signaling pathway	12	2666	7.78×10^{-7}	2.44×10^{-5}
	ko04120	KEGG	Ubiquitin mediated proteolysis	27	2666	8.83×10^{-7}	2.44×10^{-5}
	ko04068	KEGG	FoxO signaling pathway	19	2666	2.44×10^{-6}	5.06×10^{-5}
	ko04010	KEGG	MAPK signaling pathway	25	2666	2.87×10^{-6}	5.59×10^{-5}
	ko00230	KEGG	Purine metabolism	34	2666	3.55×10^{-6}	6.09×10^{-5}
	ko04144	KEGG	Endocytosis	32	2666	3.68×10^{-6}	6.09×10^{-5}
	ko04070	KEGG	Phosphatidylinositol signaling system	19	2666	5.01×10^{-6}	7.89×10^{-5}
	ko04110	KEGG	Cell cycle	25	2666	8.21×10^{-6}	0.00012
	ko04722	KEGG	Neurotrophin signaling pathway	16	2666	1.29×10^{-5}	0.00019
	ko04152	KEGG	AMPK signaling pathway	20	2666	1.57×10^{-5}	0.00021
	ko04330	KEGG	Notch signaling pathway	11	2666	2.41×10^{-5}	0.00029
	ko04668	KEGG	TNF signaling pathway	10	2666	3.23×10^{-5}	0.00035
	ko04550	KEGG	Signaling pathways regulating pluripotency of stem cells	17	2666	3.32×10^{-5}	0.00035
	ko04530	KEGG	Tight junction	16	2666	7.15×10^{-5}	0.00070
	ko04910	KEGG	Insulin signaling pathway	19	2666	7.44×10^{-5}	0.00070
	ko04370	KEGG	VEGF signaling pathway	9	2666	7.68×10^{-5}	0.00071
**module 2**	GO:0004930	Molecular Function	G-protein coupled receptor	122	713	8.99×10^{-162}	1.42×10^{-158}

			activity				
	GO:0007186	Biological Process	G-protein coupled receptor signaling pathway	123	713	2.90×10^{-156}	2.29×10^{-153}
	GO:0016021	Cellular Component	integral to membrane	161	713	5.26×10^{-92}	2.76×10^{-89}
	GO:0004983	Molecular Function	neuropeptide Y receptor activity	9	713	5.56×10^{-14}	2.19×10^{-11}
	GO:0016020	Cellular Component	membrane	63	713	3.95×10^{-13}	1.24×10^{-10}
	GO:0015991	Biological Process	ATP hydrolysis coupled proton transport	12	713	7.18×10^{-12}	1.89×10^{-9}
	GO:0004965	Molecular Function	G-protein coupled GABA receptor activity	7	713	4.95×10^{-11}	1.12×10^{-8}
	GO:0006508	Biological Process	proteolysis	36	713	8.54×10^{-11}	1.68×10^{-8}
	GO:0016500	Molecular Function	protein-hormone receptor activity	6	713	1.48×10^{-9}	2.58×10^{-7}
	GO:0008236	Molecular Function	serine-type peptidase activity	9	713	2.97×10^{-8}	4.67×10^{-6}
	GO:0015914	Biological Process	phospholipid transport	5	713	4.39×10^{-8}	5.76×10^{-6}
	GO:0004012	Molecular Function	phospholipid-translocating ATPase activity	5	713	4.39×10^{-8}	5.76×10^{-6}
	GO:0033179	Cellular Component	proton-transporting V-type ATPase, V0 domain	6	713	1.14×10^{-7}	1.38×10^{-5}
	GO:0015078	Molecular Function	hydrogen ion transmembrane transporter activity	7	713	1.75×10^{-6}	0.00020
	GO:0055114	Biological Process	oxidation-reduction process	31	713	4.94×10^{-6}	0.00052
	GO:0042246	Biological Process	tissue regeneration	4	713	6.34×10^{-6}	0.00059
	GO:0008241	Molecular Function	peptidyl-dipeptidase activity	4	713	6.34×10^{-6}	0.00059
	GO:0033178	Cellular Component	proton-transporting two-sector ATPase complex, catalytic domain	5	713	1.71×10^{-5}	0.0015
	GO:0005000	Molecular Function	vasopressin receptor activity	3	713	3.86×10^{-5}	0.0030
	GO:0004948	Molecular Function	calcitonin receptor activity	3	713	3.86×10^{-5}	0.0030
	GO:0004222	Molecular Function	metalloendopeptidase activity	9	713	8.75×10^{-5}	0.0066
	ko04080	KEGG	Neuroactive ligand-receptor interaction	32	713	9.16×10^{-32}	3.03×10^{-29}
	ko04145	KEGG	Phagosome	11	713	2.30×10^{-6}	0.00015
	ko00190	KEGG	Oxidative phosphorylation	13	713	7.01×10^{-6}	0.00039
	ko04024	KEGG	cAMP signaling pathway	12	713	1.01×10^{-5}	0.00048
	ko04721	KEGG	Synaptic vesicle cycle	8	713	2.68×10^{-5}	0.0010
	ko04966	KEGG	Collecting duct acid secretion	5	713	2.85×10^{-5}	0.0010
	ko04142	KEGG	Lysosome	12	713	3.58×10^{-5}	0.0011
**module 3**	GO:0008290	Cellular Component	F-actin capping protein complex	3	268	2.04×10^{-6}	0.0016
	GO:0051016	Biological Process	barbed-end actin filament capping	3	268	2.04×10^{-6}	0.0016
	ko04144	KEGG	Endocytosis	8	268	8.28×10^{-5}	0.026
**module 4**	GO:0006270	Biological Process	DNA replication initiation	8	254	1.77×10^{-14}	2.78×10^{-11}
	GO:0042555	Cellular Component	MCM complex	7	254	1.21×10^{-12}	9.50×10^{-10}
	GO:0003678	Molecular Function	DNA helicase activity	8	254	1.68×10^{-10}	8.83×10^{-8}
	GO:0005515	Molecular Function	protein binding	52	254	4.15×10^{-9}	1.64×10^{-6}
	GO:0006260	Biological Process	DNA replication	7	254	8.95×10^{-7}	0.00028
	ko03050	KEGG	Proteasome	10	254	1.43×10^{-11}	4.72×10^{-9}
	ko03030	KEGG	DNA replication	8	254	2.72×10^{-8}	4.49×10^{-6}
	ko04110	KEGG	Cell cycle	8	254	5.00×10^{-6}	0.00028
**module 5**	GO:0055085	Biological Process	transmembrane transport	147	150	2.85×10^{-284}	4.49×10^{-281}
	GO:0016021	Cellular Component	integral to membrane	147	150	8.44×10^{-222}	6.65×10^{-219}
	GO:0022857	Molecular Function	transmembrane transporter activity	62	150	1.92×10^{-125}	1.01×10^{-122}
	GO:0022891	Molecular Function	substrate-specific transmembrane transporter activity	28	150	1.81×10^{-57}	7.15×10^{-55}
	GO:0042626	Molecular Function	ATPase activity, coupled to transmembrane movement of substances	15	150	1.57×10^{-28}	4.95×10^{-26}
	GO:0016887	Molecular Function	ATPase activity	13	150	2.47×10^{-14}	6.48×10^{-12}
	GO:0016020	Cellular Component	membrane	28	150	4.49×10^{-14}	1.01×10^{-11}
	GO:0006810	Biological Process	transport	16	150	4.12×10^{-13}	8.12×10^{-11}
	ko02010	KEGG	ABC transporters	7	150	2.41×10^{-11}	7.96×10^{-9}
**module 6**	GO:0000786	Cellular Component	nucleosome	142	142	0	0
	GO:0006334	Biological Process	nucleosome assembly	142	142	0	0
	GO:0003677	Molecular Function	DNA binding	142	142	2.76×10^{-215}	1.45×10^{-212}
**module 7**	GO:0004252	Molecular Function	serine-type endopeptidase	13	132	3.49×10^{-12}	5.50×10^{-9}

			activity				
	GO:0006835	Biological Process	dicarboxylic acid transport	5	132	5.35×10^{-11}	2.81×10^{-8}
	GO:0017153	Molecular Function	sodium:dicarboxylate symporter activity	5	132	5.35×10^{-11}	2.81×10^{-8}
	GO:0003824	Molecular Function	catalytic activity	19	132	9.43×10^{-10}	3.72×10^{-7}
	GO:0006508	Biological Process	proteolysis	14	132	8.17×10^{-9}	2.58×10^{-6}
**module 8**	GO:0007156	Biological Process	homophilic cell adhesion	24	132	3.75×10^{-48}	5.90×10^{-45}
	GO:0016020	Cellular Component	membrane	46	132	3.21×10^{-35}	2.53×10^{-32}
	GO:0006486	Biological Process	protein glycosylation	18	132	1.29×10^{-32}	6.75×10^{-30}
	GO:0005509	Molecular Function	calcium ion binding	28	132	1.02×10^{-31}	4.02×10^{-29}
	GO:0008417	Molecular Function	fucosyltransferase activity	9	132	1.13×10^{-19}	3.55×10^{-17}
	GO:0008378	Molecular Function	galactosyltransferase activity	6	132	1.50×10^{-12}	3.93×10^{-10}
	GO:0004576	Molecular Function	oligosaccharyl transferase activity	3	132	2.41×10^{-7}	5.42×10^{-5}
	ko00601	KEGG	Glycosphingolipid biosynthesis - lacto and neolacto series	4	132	1.01×10^{-7}	3.35×10^{-5}
**module 9**	GO:0005840	Cellular Component	ribosome	65	67	4.34×10^{-159}	6.83×10^{-156}
	GO:0003735	Molecular Function	structural constituent of ribosome	65	67	5.02×10^{-157}	3.95×10^{-154}
	GO:0006412	Biological Process	translation	63	67	2.14×10^{-148}	1.12×10^{-145}
	GO:0005622	Cellular Component	intracellular	46	67	2.76×10^{-70}	1.09×10^{-67}
	GO:0019843	Molecular Function	rRNA binding	4	67	1.40×10^{-9}	4.40×10^{-7}
	ko03010	KEGG	Ribosome	57	67	7.67×10^{-130}	2.54×10^{-127}
**module 10**	GO:0016491	Molecular Function	oxidoreductase activity	8	58	9.93×10^{-8}	0.00016
	GO:0006457	Biological Process	protein folding	5	58	5.93×10^{-7}	0.00047
	GO:0008152	Biological Process	metabolic process	7	58	1.79×10^{-5}	0.0094
	GO:0006096	Biological Process	glycolysis	3	58	3.85×10^{-5}	0.015
	GO:0055114	Biological Process	oxidation-reduction process	7	58	7.39×10^{-5}	0.023
	GO:0003824	Molecular Function	catalytic activity	8	58	9.93×10^{-5}	0.026
	ko01200	KEGG	Carbon metabolism	7	58	4.07×10^{-9}	1.35×10^{-6}
	ko01230	KEGG	Biosynthesis of amino acids	4	58	1.37×10^{-5}	0.0015
	ko00030	KEGG	Pentose phosphate pathway	3	58	2.19×10^{-5}	0.0018

Table S14. 300 genes directly interacting with the IIS-PI3K-Akt-FOXO signaling pathway in the module 1.

Gene ID	Gene Annotation
Sfur-100.28	Uncharacterized protein KIAA1143-like protein
Sfur-10.195	WD repeat-containing protein 92
Sfur-103.76	Transcription initiation factor TFIID subunit 7
Sfur-107.8	Interferon-related developmental regulator 1
Sfur-108.44	ATP-dependent helicase BRM
Sfur-11.127	116 kDa U5 small nuclear ribonucleoprotein component
Sfur-1.112	Eukaryotic translation initiation factor 3 subunit M
Sfur-113.28	Splicing factor U2af 38 kDa subunit
Sfur-115.31	Vacuolar protein sorting-associated protein 4A
Sfur-117.89	SNW domain-containing protein 1
Sfur-125.52	RAC serine/threonine-protein kinase
Sfur-127.16	Actin-like protein 6A
Sfur-12.99	Activator of 90 kDa heat shock protein ATPase homolog 1
Sfur-13.96	Ribosome biogenesis protein BOP1 homolog
Sfur-14.109	Acyl-CoA-binding domain-containing protein 6
Sfur-14.118	Dual specificity mitogen-activated protein kinase kinase 3
Sfur-14.119	Arginine--tRNA ligase, cytoplasmic
Sfur-143.36	Inorganic pyrophosphatase
Sfur-1444.14	26S proteasome non-ATPase regulatory subunit 4
Sfur-145.20	tRNA (cytosine(34)-C(5))-methyltransferase
Sfur-146.10	RRP12-like protein
Sfur-15.222	Plexin-A4
Sfur-15.44	14-3-3 protein epsilon
Sfur-156.37	Coatomer subunit epsilon
Sfur-159.20	Sox21b
Sfur-170.7	N-acetyltransferase 10
Sfur-17.195	RNA-binding protein squid
Sfur-172.36	Fragile X mental retardation syndrome-related protein 1
Sfur-17.307	Mitogen-activated protein kinase 14B
Sfur-17.327	Peptide chain release factor 1-like, mitochondrial
Sfur-17.341	Caspase-1
Sfur-176.20	Protein ROP
Sfur-177.24	Methionine--tRNA ligase, cytoplasmic
Sfur-17.78	Serrate RNA effector molecule homolog
Sfur-178.5	Methyltransferase-like protein 13
Sfur-17.96	S-adenosylmethionine synthase
Sfur-18.195	Protein AATF
Sfur-182.15	Nuclear valosin-containing protein-like
Sfur-184.36	DnaJ homolog dnj-20
Sfur-189.18	Ubiquitin carboxyl-terminal hydrolase isozyme L5
Sfur-190.9	Elongator complex protein 3

Sfur-191.25	Ras association domain-containing protein 2
Sfur-19.139	Importin-11
Sfur-197.28	Protein bicaudal D
Sfur-19.87	Lon protease homolog, mitochondrial
Sfur-200.15	Exosome complex component RRP40
Sfur-200.3	rRNA 2'-O-methyltransferase fibrillarin
Sfur-20.111	Sarcolemmal membrane-associated protein
Sfur-20.149	Ras-related protein Rab-35
Sfur-20.207	Ras-like protein 3
Sfur-203.3	Peroxisomal membrane protein PEX14
Sfur-203.42	Spectrin alpha chain
Sfur-20.345	Transcriptional adapter 3
Sfur-20.346	Selenide, water dikinase
Sfur-205.4	Acetyl-CoA carboxylase
Sfur-207.25	Remodeling and spacing factor 1
Sfur-20.90	Protein 4.1 homolog
Sfur-21.120	Synembryn-A
Sfur-211.2	Angiotensin-converting enzyme (Fragment)
Sfur-21.55	Ubiquitin carboxyl-terminal hydrolase 12
Sfur-216.13	Protein FRA10AC1 homolog
Sfur-219.22	Serine hydroxymethyltransferase
Sfur-22.100	Ran-binding protein 10
Sfur-22.34	RAN GTPase-activating protein 1
Sfur-226.23	Maternal protein tudor
Sfur-226.28	3-phosphoinositide-dependent protein kinase 1
Sfur-231.50	Probable RNA-binding protein 19
Sfur-233.2	Phosphatidylinositol 3-kinase 1
Sfur-23.94	Pre-mRNA-splicing factor 38A
Sfur-240.31	Inactive peptidyl-prolyl cis-trans isomerase shutdown
Sfur-24.110	Bromodomain adjacent to zinc finger domain protein 2B
Sfur-242.27	60S ribosomal protein L28
Sfur-242.29	Cold shock domain-containing protein E1
Sfur-24.248	Replication protein A 70 kDa DNA-binding subunit
Sfur-24.257	Epidermal growth factor receptor substrate 15-like 1
Sfur-24.295	Tudor and KH domain-containing protein
Sfur-24.330	General vesicular transport factor p115
Sfur-248.4	U4/U6 small nuclear ribonucleoprotein Prp4
Sfur-248.8	60S ribosomal protein L4
Sfur-255.20	V-type proton ATPase catalytic subunit A
Sfur-269.6	Heat shock 70 kDa protein cognate 3
Sfur-269.7	E3 UFM1-protein ligase 1 homolog
Sfur-27.60	T-complex protein 1 subunit alpha
Sfur-27.95	Cytokine receptor
Sfur-2.80	GTP-binding protein 128up

Sfur-28.129	Lysine--tRNA ligase
Sfur-28.147	Nuclear pore complex protein Nup50
Sfur-284.19	Microfibrillar-associated protein 1
Sfur-284.20	Spectrin beta chain, non-erythrocytic 5
Sfur-290.10	Eukaryotic translation initiation factor 3 subunit A
Sfur-303.21	60S ribosomal protein L18
Sfur-31.129	Heterogeneous nuclear ribonucleoprotein K
Sfur-32.110	Notchless protein homolog 1
Sfur-33.103	Williams-Beuren syndrome chromosomal region 16 protein
Sfur-33.148	Replication factor C subunit 1
Sfur-33.49	ATP-dependent RNA helicase abstrakt
Sfur-347.22	Probable ATP-dependent RNA helicase DDX23
Sfur-347.4	Heterogeneous nuclear ribonucleoprotein F
Sfur-34.75	Chromatin-remodeling complex ATPase chain Iswi
Sfur-347.8	Cullin-4A
Sfur-35.128	Putative uncharacterized protein
Sfur-357.21	Cyclin-dependent kinase 10
Sfur-35.8	Splicing factor 3B subunit 3
Sfur-36.1	Transcriptional repressor CTCF
Sfur-366.11	Serine/threonine-protein phosphatase 6 regulatory subunit 3
Sfur-3.75	STIP1 homology and U box-containing protein 1
Sfur-37.64	Signal recognition particle subunit SRP72
Sfur-38.33	Peptidyl-prolyl cis-trans isomerase FKBP8
Sfur-4.137	E3 SUMO-protein ligase RanBP2
Sfur-415.4	40S ribosomal protein S26
Sfur-449.12	DNA-directed RNA polymerase I subunit rpa1
Sfur-45.109	Proteasome inhibitor PI31 subunit
Sfur-45.46	DNA polymerase alpha catalytic subunit
Sfur-464.10	Guanine nucleotide-binding protein subunit beta-2-like 1
Sfur-49.80	Mothers against decapentaplegic homolog 3
Sfur-50.260	Protein melted
Sfur-511.13	Ribosomal protein S6 kinase alpha-1
Sfur-51.62	Serine/threonine-protein kinase PRP4 homolog
Sfur-5.198	Forkhead box protein O
Sfur-5.204	Cell division cycle 5-like protein
Sfur-52.156	FACT complex subunit SSRP1
Sfur-54.28	Glucosidase 2 subunit beta
Sfur-55.107	pre-rRNA processing protein FTSJ3
Sfur-55.29	FACT complex subunit SPT16
Sfur-55.93	Chromobox protein homolog 5
Sfur-56.35	UPF0430 protein CG31712
Sfur-565.12	Aldehyde dehydrogenase, mitochondrial
Sfur-57.7	Protein tumorous imaginal discs, mitochondrial
Sfur-58.164	Tuftelin-interacting protein 11

Sfur-5.86	Casein kinase II subunit alpha
Sfur-59.127	Dipeptidyl peptidase 3
Sfur-594.3	SUMO-activating enzyme subunit 2
Sfur-59.59	40S ribosomal protein S9
Sfur-59.74	Dual specificity mitogen-activated protein kinase kinase 4
Sfur-606.4	Calcyclin-binding protein
Sfur-61.20	Peptidyl-prolyl cis-trans isomerase-like 4
Sfur-61.9	Pre-mRNA-processing factor 17
Sfur-621.10	m7GpppX diphosphatase
Sfur-62.35	Ras-like protein 1
Sfur-630.1	Chromodomain-helicase-DNA-binding protein Mi-2 homolog
Sfur-65.26	General transcription factor IIF subunit 1
Sfur-664.3	Nucleolar complex protein 3 homolog
Sfur-66.81	Sterile alpha and TIR motif-containing protein 1
Sfur-66.93	Peptidyl-prolyl cis-trans isomerase-like 2
Sfur-6.85	HMG domain-containing protein 4
Sfur-68.70	Glutamate-rich WD repeat-containing protein 1
Sfur-696.14	Zinc finger protein on ecdysone puffs
Sfur-696.2	Heat shock protein 83
Sfur-7.138	RING finger protein 113A
Sfur-72.121	Pre-mRNA-splicing factor ISY1 homolog
Sfur-72.329	Centaurin-gamma-1A
Sfur-72.386	RING finger protein unkempt
Sfur-72.474	Tuberin
Sfur-72.483	MRG/MORF4L-binding protein
Sfur-72.510	Heterogeneous nuclear ribonucleoprotein A1, A2/B1 homolog
Sfur-77.62	Insulin receptor substrate 1
Sfur-77.6	COP9 signalosome complex subunit 2
Sfur-783.2	Probable H/ACA ribonucleoprotein complex subunit 1
Sfur-79.20	Heat shock 70 kDa protein cognate 5
Sfur-7.92	Phosphatidylinositol 3-kinase regulatory subunit alpha
Sfur-80.31	Negative elongation factor B
Sfur-80.73	F-box only protein 28
Sfur-81.46	Importin-7
Sfur-81.48	Peptide transporter family 1
Sfur-8.165	YTH domain-containing family protein 2
Sfur-84.32	Ubiquitin carboxyl-terminal hydrolase 36
Sfur-86.73	Enhancer of mRNA-decapping protein 4
Sfur-87.18	Cactin
Sfur-87.49	Splicing factor 3A subunit 3
Sfur-92.26	Probable 28S rRNA (cytosine-C(5))-methyltransferase
Sfur-9.332	DNA damage-binding protein 1
Sfur-95.51	Phosphatidylinositol 3,4,5-trisphosphate 3-phosphatase cnrN
Sfur-97.98	Pre-mRNA-processing factor 19

Sfur-45.89	Insulin-like receptor
Sfur-149.4	60S ribosomal protein L35
Sfur-168.45	Multidrug resistance-associated protein 5
Sfur-24.124	Rho GTPase-activating protein 190
Sfur-265.9	Aspartate--tRNA ligase, mitochondrial
Sfur-62.25	Protein polybromo-1
Sfur-63.96	Chromodomain-helicase-DNA-binding protein 1
Sfur-110.17	Protein strawberry notch
Sfur-114.28	Nuclear speckle splicing regulatory protein 1
Sfur-116.41	Glycine-rich protein GRP33
Sfur-128.8	Coiled-coil domain-containing protein 25
Sfur-139.29	Neurofilament heavy polypeptide
Sfur-142.18	Vacuole membrane protein 1
Sfur-15.41	Phosphatidylinositol 4-phosphate 5-kinase type-1 alpha
Sfur-15.92	Round spermatid basic protein 1-like protein
Sfur-16.127	Vacuolar protein sorting-associated protein 53 homolog
Sfur-162.31	RNA-binding protein 26
Sfur-169.12	Programmed cell death protein 5
Sfur-171.17	Serine/threonine-protein kinase OSR1
Sfur-17.120	Structural maintenance of chromosomes protein 6
Sfur-171.24	Uncharacterized protein
Sfur-181.24	Chromodomain-helicase-DNA-binding protein 7
Sfur-18.228	Tigger transposable element-derived protein 6
Sfur-18.236	DnaJ homolog subfamily C member 9
Sfur-185.45	F-box/WD repeat-containing protein 1A
Sfur-192.22	Coiled-coil domain-containing protein 137
Sfur-201.11	Quinone oxidoreductase
Sfur-20.8	G protein-coupled receptor kinase 2
Sfur-208.30	Ribosomal RNA processing protein 36 homolog
Sfur-21.135	Protein wings apart-like
Sfur-21.136	Macoilin
Sfur-21.38	ADP-ribosylation factor GTPase-activating protein 3
Sfur-217.38	U4/U6.U5 small nuclear ribonucleoprotein 27 kDa protein
Sfur-24.196	G2/M phase-specific E3 ubiquitin-protein ligase
Sfur-24.212	Zinc finger protein 729
Sfur-24.217	F-BAR domain only protein 2
Sfur-26.50	Heterogeneous nuclear ribonucleoprotein U-like protein 1
Sfur-27.85	Inositol 1,4,5-trisphosphate receptor
Sfur-28.131	Uncharacterized protein (Fragment)
Sfur-283.12	Nuclear migration protein nudC
Sfur-283.4	ESF1 homolog
Sfur-29.125	E3 ubiquitin-protein ligase HUWE1
Sfur-305.19	Exportin-4
Sfur-31.151	General transcription factor IIH subunit 4

Sfur-314.14	RanBP-type and C3HC4-type zinc finger-containing protein 1
Sfur-316.7	Cap-specific mRNA (nucleoside-2'-O-)-methyltransferase 1
Sfur-327.11	Transitional endoplasmic reticulum ATPase TER94
Sfur-330.15	Uncharacterized protein
Sfur-34.10	Probable ATP-dependent RNA helicase DDX60
Sfur-356.17	ATP-binding cassette sub-family F member 1
Sfur-4.114	Beta-catenin-like protein 1
Sfur-4.119	Transmembrane and coiled-coil domain-containing protein 4
Sfur-42.105	Biorientation of chromosomes in cell division protein 1
Sfur-42.52	Uncharacterized protein
Sfur-43.100	Putative uncharacterized protein
Sfur-48.91	Protein Peter pan
Sfur-51.4	Protein croquemort
Sfur-540.11	F-box/LRR-repeat protein 7
Sfur-55.118	Cold shock domain-containing protein CG9705
Sfur-55.27	Protein lin-37 homolog
Sfur-55.35	Cerebellar degeneration-related protein 2-like
Sfur-553.8	Protein bric-a-brac 2
Sfur-55.41	Uncharacterized protein
Sfur-56.59	Monocarboxylate transporter 14
Sfur-56.78	Kinesin-like protein unc-104
Sfur-57.63	GPALPP motifs-containing protein 1
Sfur-579.4	Zinc finger protein 208
Sfur-59.21	Uncharacterized protein (Fragment)
Sfur-60.65	Cleavage and polyadenylation specificity factor subunit 2
Sfur-618.3	5'-3' exoribonuclease 2
Sfur-6.256	Zinc finger CCCH domain-containing protein 11A
Sfur-64.3	Structural maintenance of chromosomes protein 3
Sfur-6.74	Putative extracellular matrix-associated peroxidase
Sfur-70.66	Poly(A) polymerase alpha
Sfur-70.67	H/ACA ribonucleoprotein complex subunit 4
Sfur-71.79	DNA polymerase delta subunit 3
Sfur-72.275	Leucine-rich repeat protein soc-2 homolog
Sfur-724.1	SAP domain-containing ribonucleoprotein
Sfur-72.505	Luc7-like protein 3
Sfur-72.55	Ubiquitin carboxyl-terminal hydrolase 3
Sfur-73.5	RNA-directed DNA polymerase from mobile element jockey
Sfur-73.57	Transducin beta-like protein 2
Sfur-74.212	Nucleoprotein TPR
Sfur-7.76	Sister chromatid cohesion protein PDS5 homolog B
Sfur-78.59	Casein kinase I isoform epsilon
Sfur-78.76	Structural maintenance of chromosomes protein 1A
Sfur-8.164	60S ribosomal protein L30
Sfur-85.11	Dynactin subunit 1

Sfur-85.62	DNA-directed RNA polymerase II subunit RPB2
Sfur-93.2	tRNA-splicing ligase RtcB homolog 1
Sfur-9.328	Bromodomain adjacent to zinc finger domain protein 1A
Sfur-95.46	ATP-dependent RNA helicase DHX36
Sfur-303.32	85/88 kDa calcium-independent phospholipase A2
Sfur-6.16	Vacuolar protein sorting-associated protein 13D
Sfur-217.31	Protein hu-li tai shao
Sfur-58.105	Tyrosine-protein kinase CSK
Sfur-631.3	BUD13 homolog
Sfur-283.18	Copper-transporting ATPase 1
Sfur-201.32	Muscle M-line assembly protein unc-89
Sfur-97.72	Protein pelota
Sfur-55.105	Cytochrome c oxidase subunit 6A1, mitochondrial
Sfur-147.49	Probable ATP-dependent RNA helicase ddx17
Sfur-381.9	Eukaryotic translation initiation factor 3 subunit I
Sfur-76.16	Ubiquitin carboxyl-terminal hydrolase 34
Sfur-50.367	Rho guanine nucleotide exchange factor 10
Sfur-95.38	60S ribosomal protein L27
Sfur-8.171	60S ribosomal protein L8
Sfur-24.178	Plasminogen activator inhibitor 1 RNA-binding protein
Sfur-66.2	60S ribosomal protein L13a
Sfur-229.26	Protein VPRBP
Sfur-1884.1	Myelin expression factor 2
Sfur-375.14	La-related protein 1
Sfur-107.88	Unconventional myosin-IXa
Sfur-234.21	HIG1 domain family member 2A, mitochondrial
Sfur-63.30	Vascular endothelial growth factor receptor 1
Sfur-723.8	Histone deacetylase 7
Sfur-24.175	Ribosome-binding protein 1
Sfur-745.2	Zinc finger protein 37 homolog
Sfur-617.10	Claspin
Sfur-216.3	Transposon Ty3-I Gag-Pol polyprotein
Sfur-196.9	SURP and G-patch domain-containing protein 1
Sfur-85.8	Carotenoid isomerooxygenase
Sfur-70.59	Rho guanine nucleotide exchange factor 12
Sfur-189.22	Uncharacterized protein
Sfur-445.11	Uncharacterized protein
Sfur-276.15	WD repeat-containing protein 35
Sfur-400.2	Insulin-like receptor

Table S15. 45 WDRGs differentially expressed between two wing morphs.

Functional Category	Gene ID	Gene symbol	Gene Annotation
Translational initiation	Sfur-1.112	*eif3m*	Eukaryotic translation initiation factor 3 subunit M
	Sfur-381.9	*eif3i*	Eukaryotic translation initiation factor 3 subunit I
Ribosome biogenesis and assembly	Sfur-13.96	*bop1*	Ribosome biogenesis protein BOP1 homolog
	Sfur-146.10	*rrp12*	RRP12-like protein
	Sfur-231.50	*rbm19*	Probable RNA-binding protein 19
	Sfur-449.12	*polr1a*	DNA-directed RNA polymerase I subunit rpa1
	Sfur-68.70	*grwd1*	Glutamate-rich WD repeat-containing protein 1
	Sfur-92.26	*nop2*	Probable 28S rRNA (cytosine-C(5))-methyltransferase
	Sfur-18.195	*aatf*	Protein AATF
Ribosomal proteins	Sfur-242.27	*rpl28*	60S ribosomal protein L28
	Sfur-248.8	*rpl4*	60S ribosomal protein L4
	Sfur-303.21	*rpl18*	60S ribosomal protein L18
	Sfur-149.4	*rpl35*	60S ribosomal protein L35
	Sfur-8.164	*rpl30*	60S ribosomal protein L30
	Sfur-95.38	*rpl27*	60S ribosomal protein L27
	Sfur-8.171	*rpl8*	60S ribosomal protein L8
	Sfur-59.59	*rps9*	40S ribosomal protein S9
rRNA processing and modification	Sfur-170.7	*nat10*	N-acetyltransferase 10
	Sfur-200.3	*fbl*	rRNA 2'-O-methyltransferase fibrillarin
	Sfur-55.107	*ftsj3*	pre-rRNA processing protein FTSJ3
	Sfur-70.67	*dkc1*	H/ACA ribonucleoprotein complex subunit 4
Growth, cell proliferation, cell cycle and development	Sfur-159.20	*sox21b*	Sox21b
	Sfur-17.195	*sqd*	RNA-binding protein squid
	Sfur-226.23	*tudor*	Maternal protein tudor
	Sfur-283.12	*nudc*	Nuclear migration protein nudC
	Sfur-48.91	*ppan*	Protein Peter pan
	Sfur-72.55	*usp3*	Ubiquitin carboxyl-terminal hydrolase 3
Signal transduction	Sfur-226.28	*pdk1*	3-phosphoinositide-dependent protein kinase 1
	Sfur-63.30	*vegfr1*	Vascular endothelial growth factor receptor 1
	Sfur-464.10	*rack1*	Guanine nucleotide-binding protein subunit beta-2-like 1
	Sfur-32.110	*nle1*	Notchless protein homolog 1

**Functional Category**	**Gene ID**	**Gene symbol**	**Gene Annotation**
Metabolism	Sfur-17.96	*mat*	S-adenosylmethionine synthase
	Sfur-565.12	*aldh2*	Aldehyde dehydrogenase, mitochondrial
	Sfur-219.22	*shmt*	Serine hydroxymethyltransferase
protein transport, protein folding and protein degradation	Sfur-269.6	*hsc70-3*	Heat shock 70 kDa protein cognate 3
	Sfur-3.75	*stub1*	STIP1 homology and U box-containing protein 1
	Sfur-37.64	*srp72*	Signal recognition particle subunit SRP72
	Sfur-35.128	*litaf*	Lipopolysaccharide Induced TNF Factor
	Sfur-81.48	*pept1*	Peptide transporter family 1
Others	Sfur-216.13	*fra10ac1*	Protein FRA10AC1 homolog
	Sfur-7.138	*rnf113a*	RING finger protein 113A
	Sfur-283.4	*esf1*	ESF1 homolog
	Sfur-201.32	*unc-89*	Muscle M-line assembly protein unc-89
	Sfur-234.21	*higd2a*	HIG1 domain family member 2A, mitochondrial
	Sfur-284.20	*sptbn5*	Spectrin beta chain, non-erythrocytic 5

Table S16. 11 WDRGs differentially expressed between two wing morphs and identified as FOXO target genes.

Gene ID	Gene symbol	Gene Annotation
Sfur-1.112	*eif3m*	Eukaryotic translation initiation factor 3 subunit M
Sfur-170.7	*nat10*	N-acetyltransferase 10
Sfur-200.3	*fbl*	rRNA 2'-O-methyltransferase fibrillarin
Sfur-248.8	*rpl4*	60S ribosomal protein L4
Sfur-284.20	*sptbn5*	Spectrin beta chain, non-erythrocytic 5
Sfur-35.128	*litaf*	Putative uncharacterized protein
Sfur-3.75	*stub1*	STIP1 homology and U box-containing protein 1
Sfur-464.10	*rack1*	Guanine nucleotide-binding protein subunit beta-2-like 1
Sfur-68.70	*grwd1*	Glutamate-rich WD repeat-containing protein 1
Sfur-283.12	*nudc*	Nuclear migration protein nudC
Sfur-8.164	*rpl30*	60S ribosomal protein L30

Table S17. Primers for dsRNA preparation and qRT-PCR analysis.

Primer Name	Sequence (5'-3')	Purpose
InR1-RNAi-F	T7-TTGCGAAGAACCATTAAGTT	*InR1* dsRNA synthesis
InR1-RNAi-R	T7-TCTGATACTACATCGGCATT	
foxo-RNAi-F	T7-ATCCAACACTTGGCCACTTC	*foxo* dsRNA synthesis
foxo-RNAi-R	T7-TCTCGAATTTGGACGTTTCC	
rack1-RNAi-F	T7-GAGTCGAGCTATGGAGTGCC	*rack1* dsRNA synthesis
rack1-RNAi-R	T7-ACCTGACACAGGAAACCCAG	
vegfr1-RNAi-F	T7-AATGGAAAGACCTGCCAATG	*vegfr1* dsRNA synthesis
vegfr1-RNAi-R	T7-ATGTTGAGGTAGCCTGTGGG	
grwd1-RNAi-F	T7-ATCATGCTTCACAATGCCAA	*grwd1* dsRNA synthesis
grwd1-RNAi-R	T7-CGAAGACAAAGAGCGGTTTC	
nop2-RNAi-F	T7-AGTACCAATCGGTGCCACTC	*nop2* dsRNA synthesis
nop2-RNAi-R	T7-AGCTTCACATCGCGATTCTT	
eif3m-RNAi-F	T7-CAGCCAAAGTGATGCAAGAA	*eif3m* dsRNA synthesis
eif3m-RNAi-R	T7-GATGAAAGCTTCGACTTGGC	
gfp-RNAi-F	T7-ACGTAAACGGCCACAAGTTC	*gfp* dsRNA synthesis
gfp-RNAi-R	T7-TGTTCTGCTGGTAGTGGTCG	
Q*InR1*-F	GTAAACCACACCCAGCCAAT	qRT-PCR for *InR1*
Q*InR1*-R	CCGGGATATGCAGTGAGTCT	
Q*foxo*-F	TGCGTCTGCAACACTCCTAA	qRT-PCR for *foxo*
Q*foxo*-R	GACTGCCCTCCATGCTGA	
Q*rack1*-F	CAAGGAAATGGTGGATGA	qRT-PCR for *rack1*
Q*rack1*-R	CAGAGTAGCCAGCGAAAA	
Q*vegfr1*-F	TTCGTGGAAATGGTATCGTC	qRT-PCR for *vegfr1*
Q*vegfr1*-R	ATCTTGACATCGTTGAGGGA	
Q*grwd1*-F	ACCGCTCTTTGTCTTCGC	qRT-PCR for *grwd1*
Q*grwd1*-R	TGCCCTCATTTGGTTTCC	
Q*nop2*-F	TGATGGATTTCTTGGAGGCT	qRT-PCR for *nop2*
Q*nop2*-R	GGCACCGATTGGTACTGG	
Q*eif3m*-F	TCTATGCTGGTGTTGGTT	qRT-PCR for *eif3m*
Q*eif3m*-R	CCTGTCTGGCAATCTGTA	
TnI-L-F	ATGGCGGACGATGAGAAAAGACGCATTGATGAG	PCR for longer isoform of *TnI*
TnI-L-R	TCAAGTATTCTTACGTTCAGCTGGGGGAGCCTC	
TnI-S-F	ATGGCGGACGATGAGAAAAGAC	PCR for shorter isoform of *TnI*
TnI-S-R	CTTCTTCTCGTCCTTCTTTGAC	
